# Supplementary figures and images for: Task-evoked activity quenches neural correlations and variability across cortical areas
Source: PLoS Comput Biol. 2020 Aug 3;16(8):e1007983. doi: 10.1371/journal.pcbi.1007983 (PMC7425988; doi:10.1371/journal.pcbi.1007983)

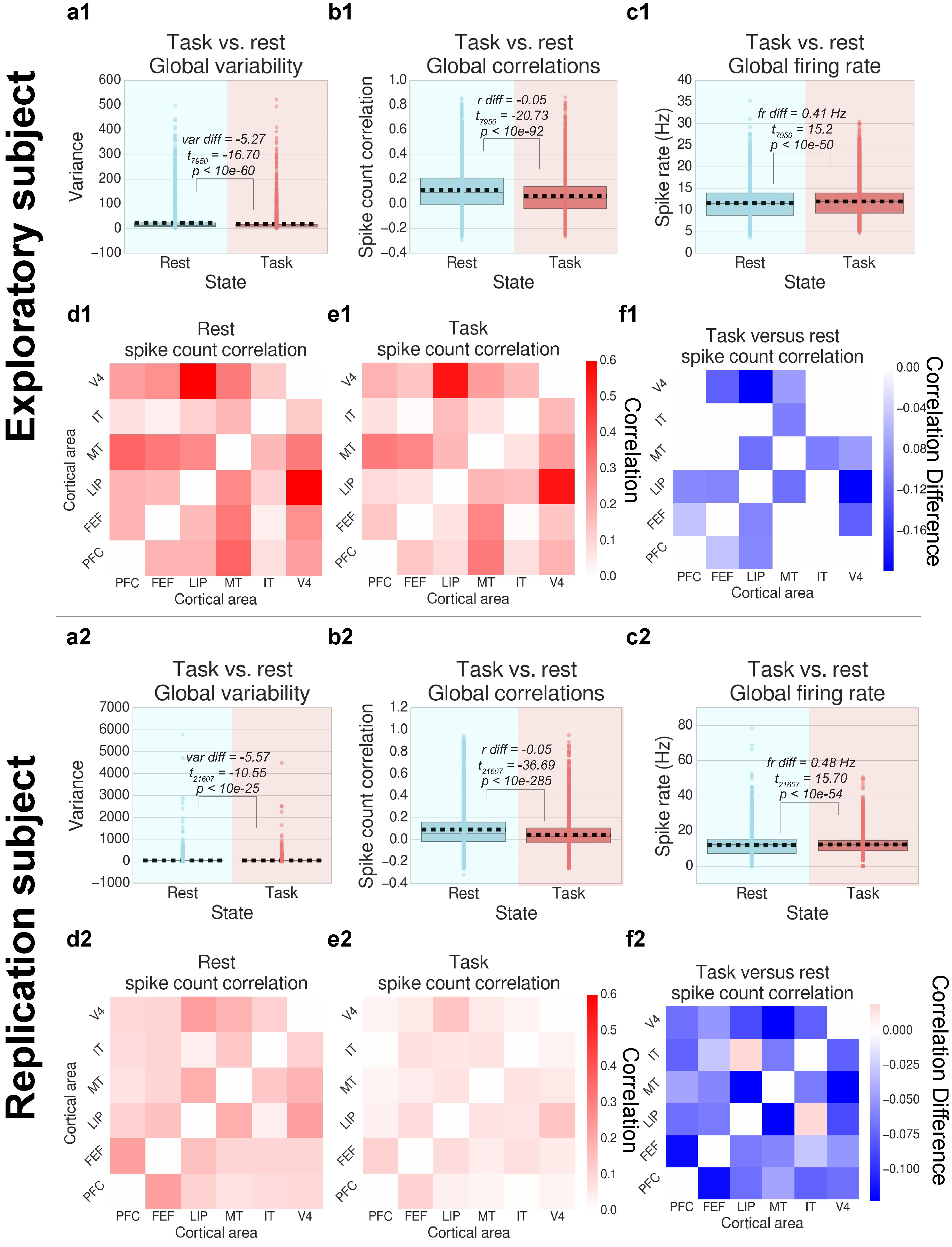

Supplement: S2 Fig — We analyzed the variability across time points (within trial) during ITIs and task cue periods to evaluate whether correlation and variability quenching also occurred on a moment-to-moment basis (i.e., faster timescale). Task cue intervals and ITIs were matched to have equivalent time points on a trial-by-trial basis. a1,a2) Global variability across the two states (estimated using the variance across time points) between task and rest state windows. b1,b2) We then calculated the global spike count correlation between the exact same task cue intervals with equivalent rest intervals between all pairs of recorded brain regions. (Spike rates were averaged within each cortical area.) c1,c2) We also calculated the global firing rate (averaged across all recording areas) during the task interval and rest interval. d1-f1,d2-f2) For each pair of brain regions, we visualize the spike count correlation matrices between each recording site for the averaged rest, task, and the differences between task versus rest state spike count correlation. For panels d-f, plots were thresholded and tested for multiple comparisons using an FDR-corrected p<0.05 threshold. Boxplots indicate the interquartile range of the distribution, dotted black line indicates the mean, grey line indicates the median, and the distribution is visualized using a strip plot. (TIF) [file pcbi.1007983.s002.tif]

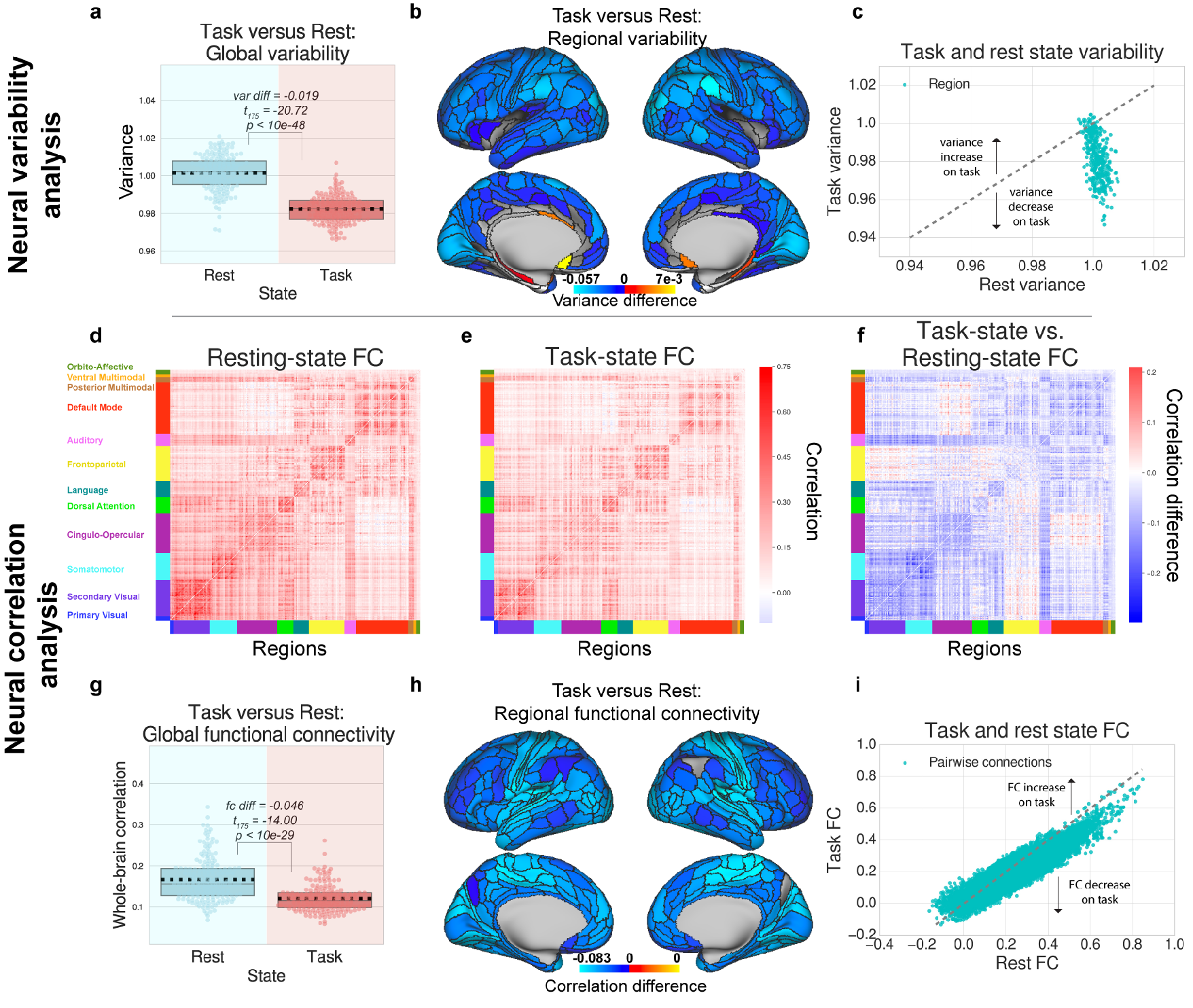

Supplement: S4 Fig — We successfully replicated results from Fig 3 using our held-out cohort of 176 subjects. a) We first compared the global variability during task and rest states, which is averaged across all brain regions, and then b) computed the task- versus rest-state variability for each brain region. c) Scatter plot depicting the variance of each parcel during task states (y-axis) and rest states (x-axis). Dotted grey line denotes no change between rest and task states. d) We next compared the correlation matrices for resting state blocks with (e) task state blocks, and (f) computed the task- versus rest-state correlation matrix difference. g) We found that the average FC between all pairs of brain regions is significantly reduced during task state. h) We found that the average correlation for each brain region, decreased for each brain region during task state. i) Scatter plot depicting the FC (correlation values) of each pair of parcels during task states (y-axis) and rest states (x-axis). Dotted grey line denotes no change between rest and task states. For panels b-f, and h, plots were tested for multiple comparisons using an FDR-corrected p<0.05 threshold. Boxplots indicate the interquartile range of the distribution, dotted black line indicates the mean, grey line indicates the median, and the distribution is visualized using a swarm plot. (TIF) [file pcbi.1007983.s004.tif]

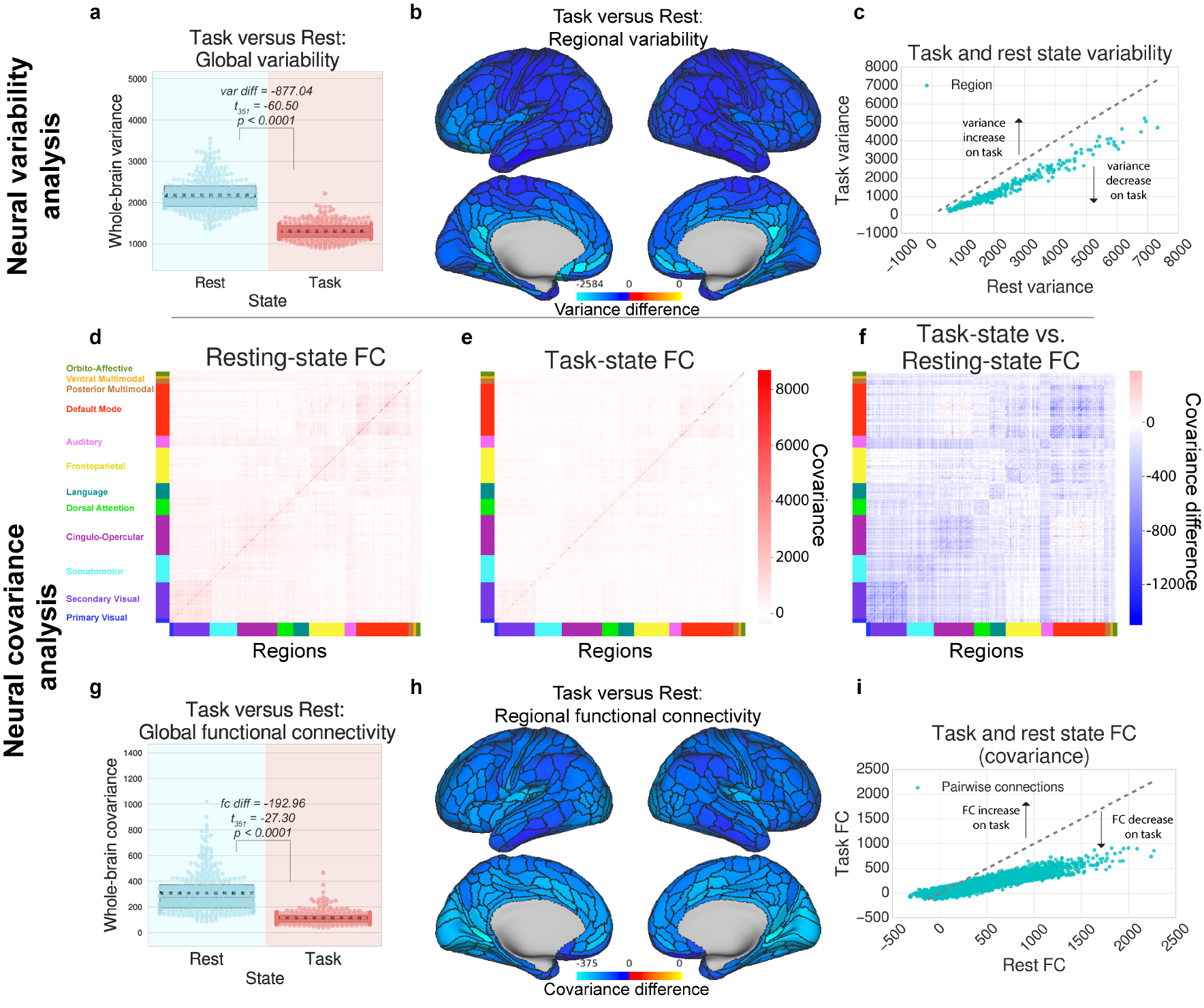

Supplement: S5 Fig — Variance and covariance decreased during task states in human fMRI data. We successfully replicated results from Fig 3 using, but without z-normalizing the time series (and using covariance instead of correlation). The combination of reduced correlations (Fig 3) and covariance measures suggested that shared signal dynamics is reduced from task to rest [31–33]. a) We first compared the global variability during task and rest states, which is averaged across all brain regions, and then b) computed the task- versus rest-state variability for each brain region. c) Scatter plot depicting the variance of each parcel during task states (y-axis) and rest states (x-axis). Dotted grey line denotes no change between rest and task states. d) We next compared the covariance matrices for resting state blocks with (e) task state blocks, and (f) computed the task- versus rest-state covariance matrix difference. g) We found that the average covariance between all pairs of brain regions is significantly reduced during task state. h) We found that the average covariance for each brain region, decreased for each brain region during task state. i) Scatter plot depicting the FC (covariance values) of each pair of parcels during task states (y-axis) and rest states (x-axis). Dotted grey line denotes no change between rest and task states. For panels b-f, and h, plots were tested for multiple comparisons using an FDR-corrected p<0.05 threshold. Boxplots indicate the interquartile range of the distribution, dotted black line indicates the mean, grey line indicates the median, and the distribution is visualized using a swarm plot. (TIF) [file pcbi.1007983.s005.tif]

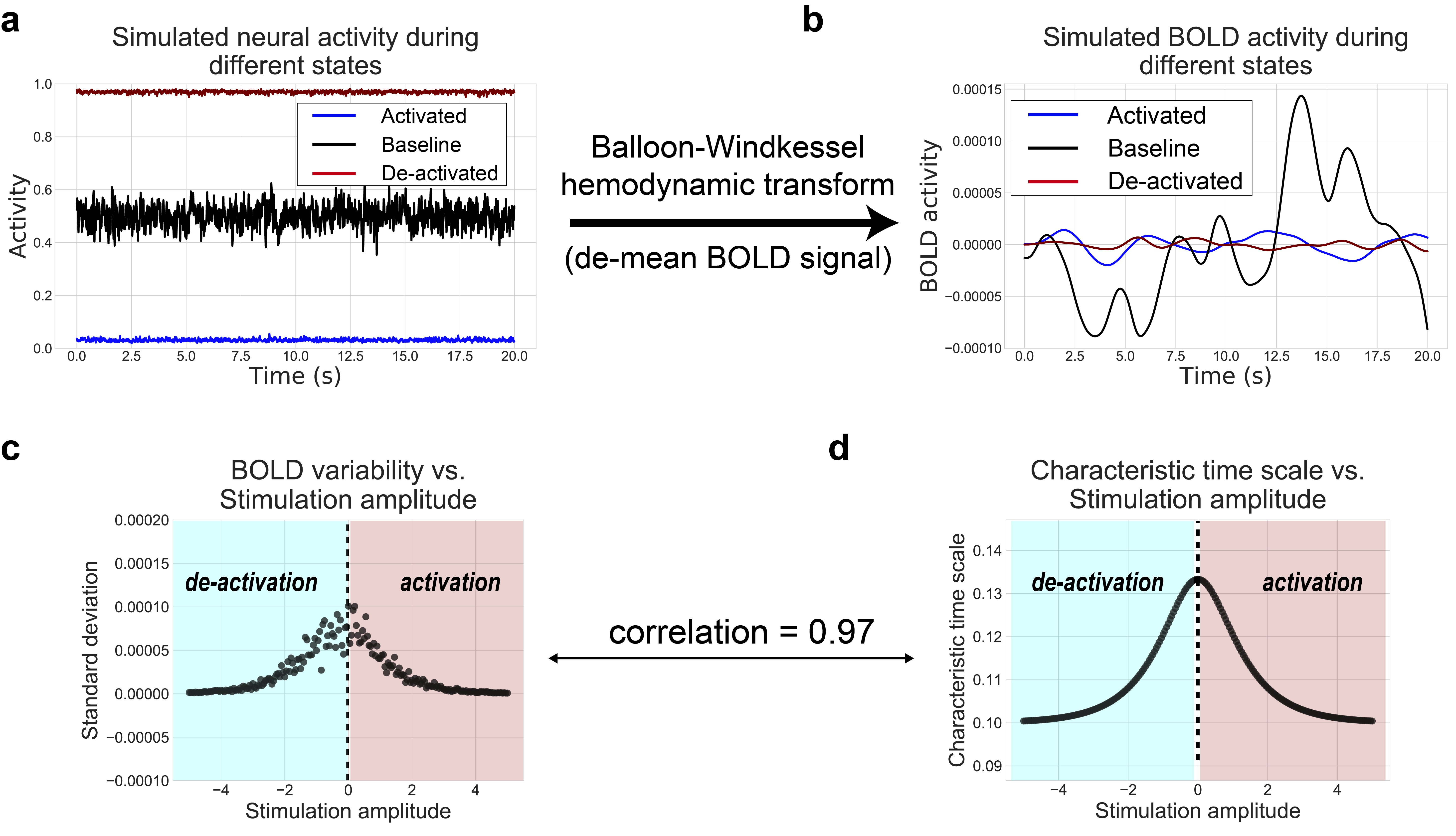

Supplement: S6 Fig — a) We simulated the neural mass model under the same three stimulus conditions (de-activated, baseline, and activated states) as in Fig 7A. b) We subsequently applied the Balloon-Windkessel transformation to the simulated neural activity, a nonlinear transformation from neural activity to the fMRI BOLD signal [44]. Notably, the transformation assumes a nonlinear transformation of the normalized deoxyhemoglobin content, normalized blood inflow, resting oxygen extraction fraction, and the normalized blood volume. All BOLD signals were de-meaned such that it is possible to visually compare the time series variance of each stimulus condition. c) We simulated BOLD activity under a range of stimulus conditions and calculated the standard deviation of each time series. d) We calculated the rank correlation of the standard deviation of the BOLD signal across stimulus conditions with the characteristic time scale at each condition. (TIF) [file pcbi.1007983.s006.tif]

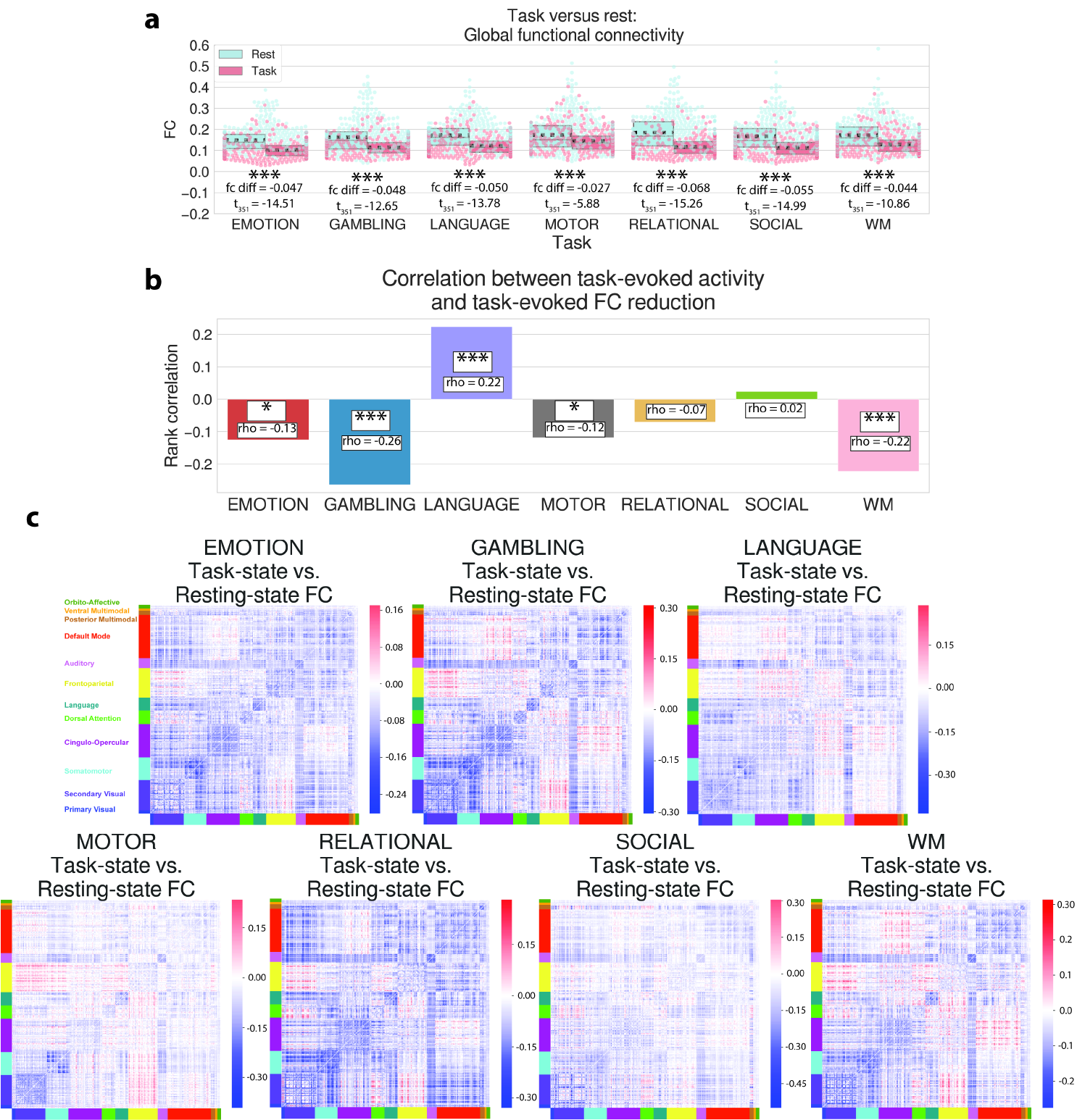

Supplement: S9 Fig — a) This panel is identical to the analysis performed in Fig 3G, except that it was performed on each HCP task separately. Whole-brain FC, averaged across all pairs of regions, was reduced for 7/7 of the HCP tasks. b) This panel is identical to the analysis performed in S3 Fig, except that the spatial correlation was performed on each HCP task separately (and is visualized as a bar plot). Regional task-evoked FC was significantly negatively correlated with the magnitude of task-evoked activation (absolute value) for 4/7 of the HCP tasks. All analyses (in panels A and B) were corrected for multiple comparisons using FDR correction. (*** = FDR-corrected p<0.0001; ** = FDR-corrected p<0.01; * = FDR-corrected p<0.05). c) Task- versus rest-state FC analysis for each of the 7 HCP tasks separately. (This figure is identical to Fig 3f, except that the statistics were performed on each task separately.) Though whole-brain FC differences from task to rest are different for each task, there are mostly FC decreases during task state relative to rest state. Boxplots indicate the interquartile range of the distribution, dotted black line indicates the mean, and the distribution is visualized using a swarm plot. (TIF) [file pcbi.1007983.s009.tif]

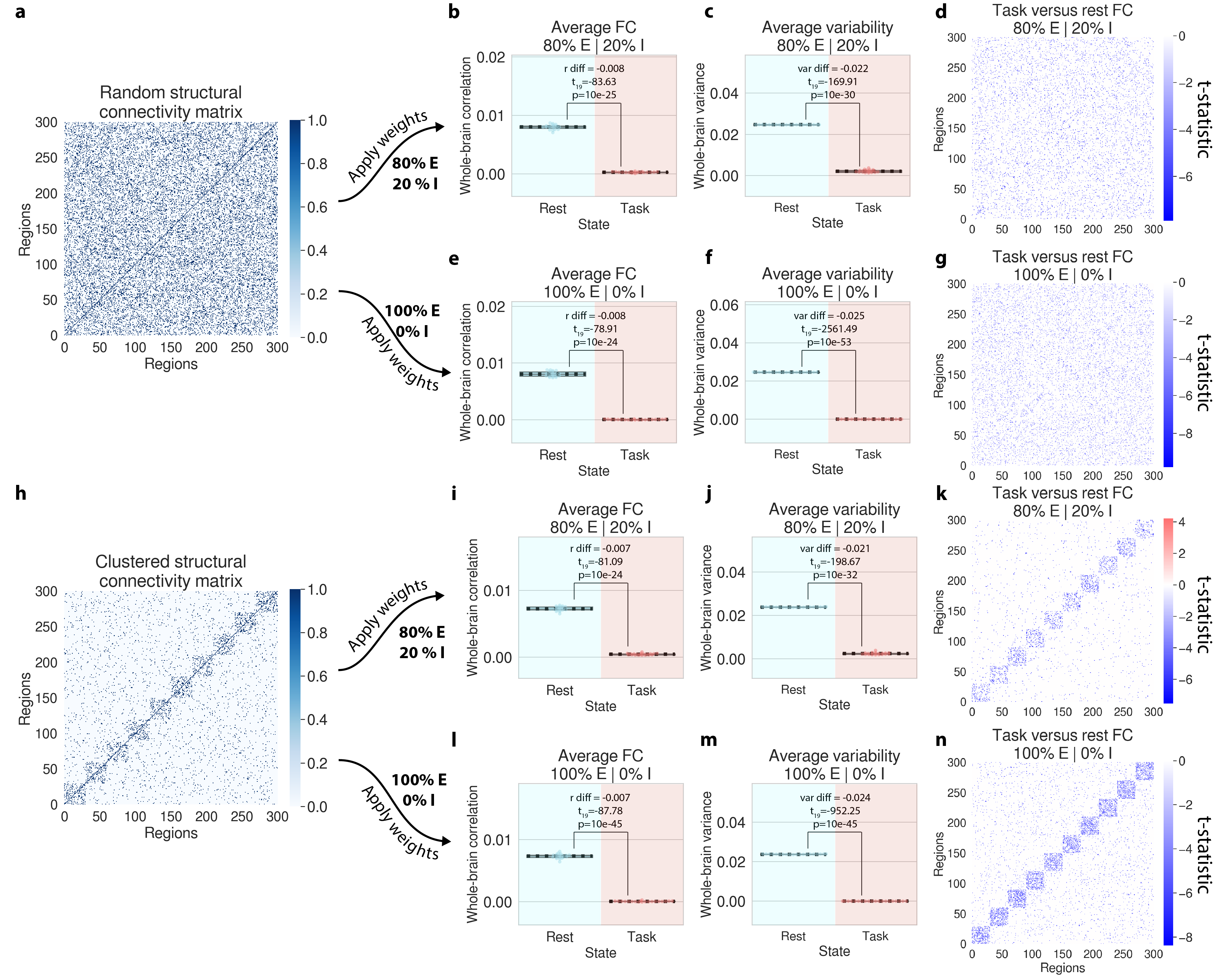

Supplement: S11 Fig — For each structural connectivity matrix, we randomly sampled synaptic weights from a normal distribution with either 100% E connections (given evidence that most long-range connections are excitatory, μ = 1.0, σ = 0.2 [42]), or 80% E and 20% I connections (μ = 1.0, σ = 1.2). For each network model (4 in total), we simulated 20 subjects for 10 seconds each (100ms sampling rate). For simplicity, during the task state, all units were stimulated with a fixed input. a) Random structural connectivity matrix (20% connectivity density) for an example subject. b) The average across all pairwise correlations during the rest and task states for the network model with 80% E and 20% I connections. The rest state exhibits higher correlations than the task state. c) The variability (variance across time) averaged across brain regions during the rest and task states for the network model with 80% E and 20% I connections. The rest state exhibits higher variability than the task state. d) The task minus rest FC matrix (correlation difference) between all 300 regions. Correlations decreased from rest to task states. e-g) The same analyses as b-d, but using only excitatory connections only. h) Clustered structural connectivity matrix (10 communities, 20% within-community density, 3% out-of-community density). i-k) The same analyses as b-d, but using the clustered connectivity matrix with 80% E and 20% I connections. l-n) The same analyses as b-d, but using the clustered connectivity matrix with 100% E connections. Boxplots indicate the interquartile range of the distribution, dotted black line indicates the mean, and the distribution is visualized using a swarm plot. Plots d, g, k, n, were corrected for multiple comparisons and thresholded using an FDR-corrected p<0.05. (TIF) [file pcbi.1007983.s011.tif]

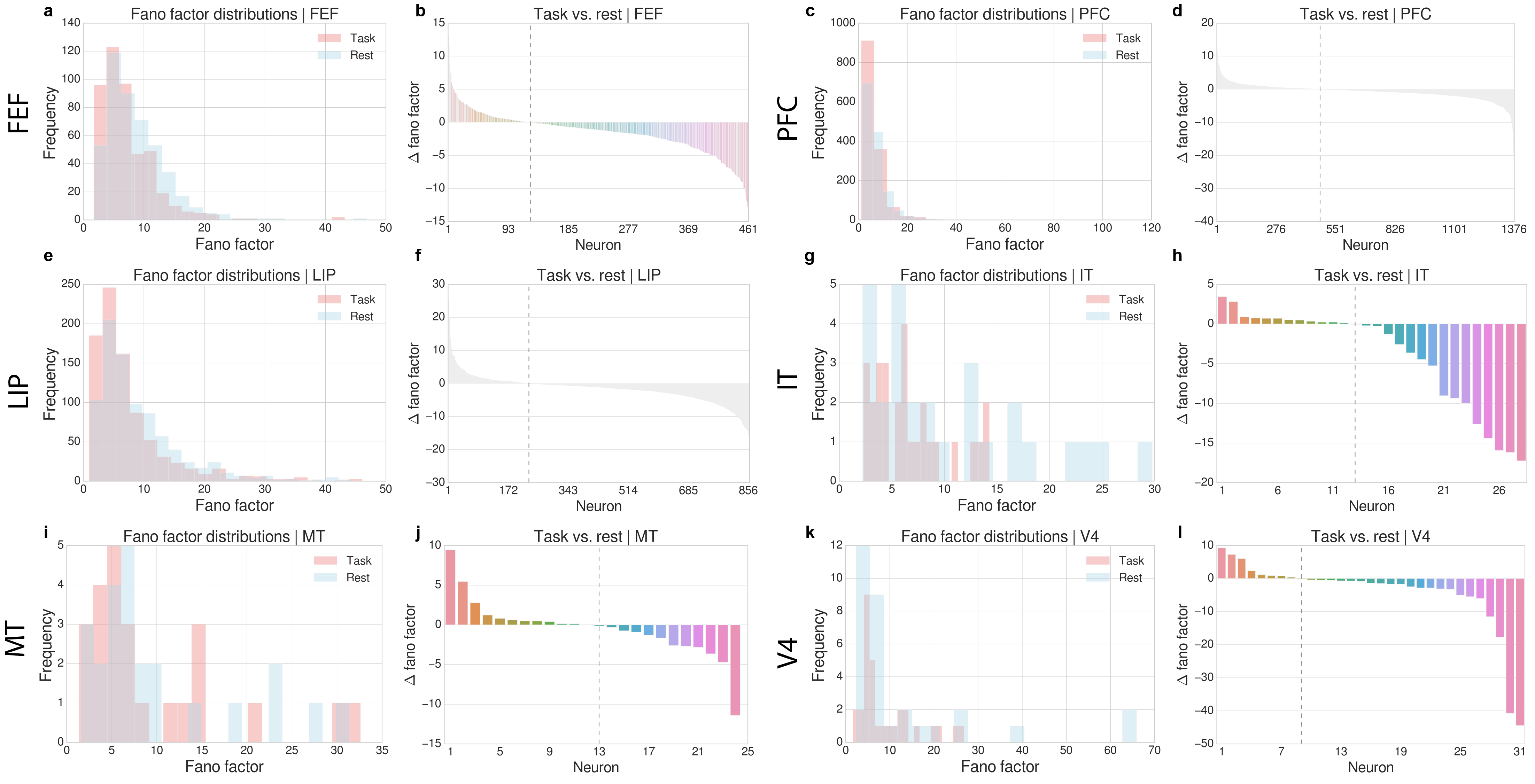

Supplement: S12 Fig — (This is not a mean-field analysis.) a) The distribution of fano factor across all neurons in FEF (from all recording sessions) for the rest (ITI) and task state (cue) periods. b) For each individual neuron in FEF, we calculated the change in fano factor from the rest to task state period. c,d) Same as a, b, but for PFC. e,f) Same as a, b, but for LIP. g,h) Same as a, b, but for IT. i,j) Same as a, b, but for MT. k,l) Same as a, b, but for V4. (TIF) [file pcbi.1007983.s012.tif]

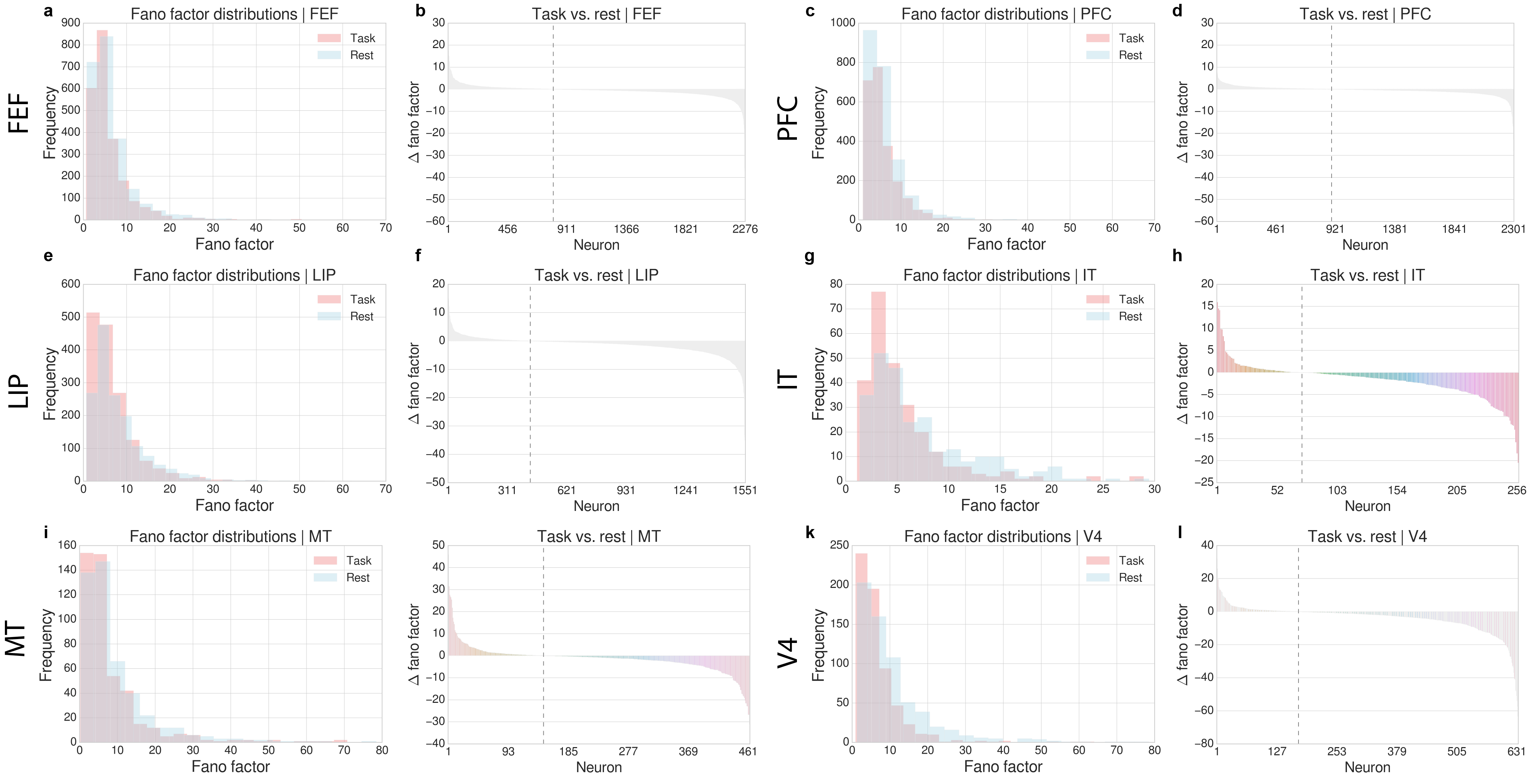

Supplement: S13 Fig — (This is not a mean-field analysis.) a) The distribution of fano factor across all neurons in FEF (from all recording sessions) for the rest (ITI) and task state (cue) periods. b) For each individual neuron in FEF, we calculated the change in fano factor from the rest to task state period. c,d) Same as a, b, but for PFC. e,f) Same as a, b, but for LIP. g,h) Same as a, b, but for IT. i,j) Same as a, b, but for MT. k,l) Same as a, b, but for V4. (TIF) [file pcbi.1007983.s013.tif]

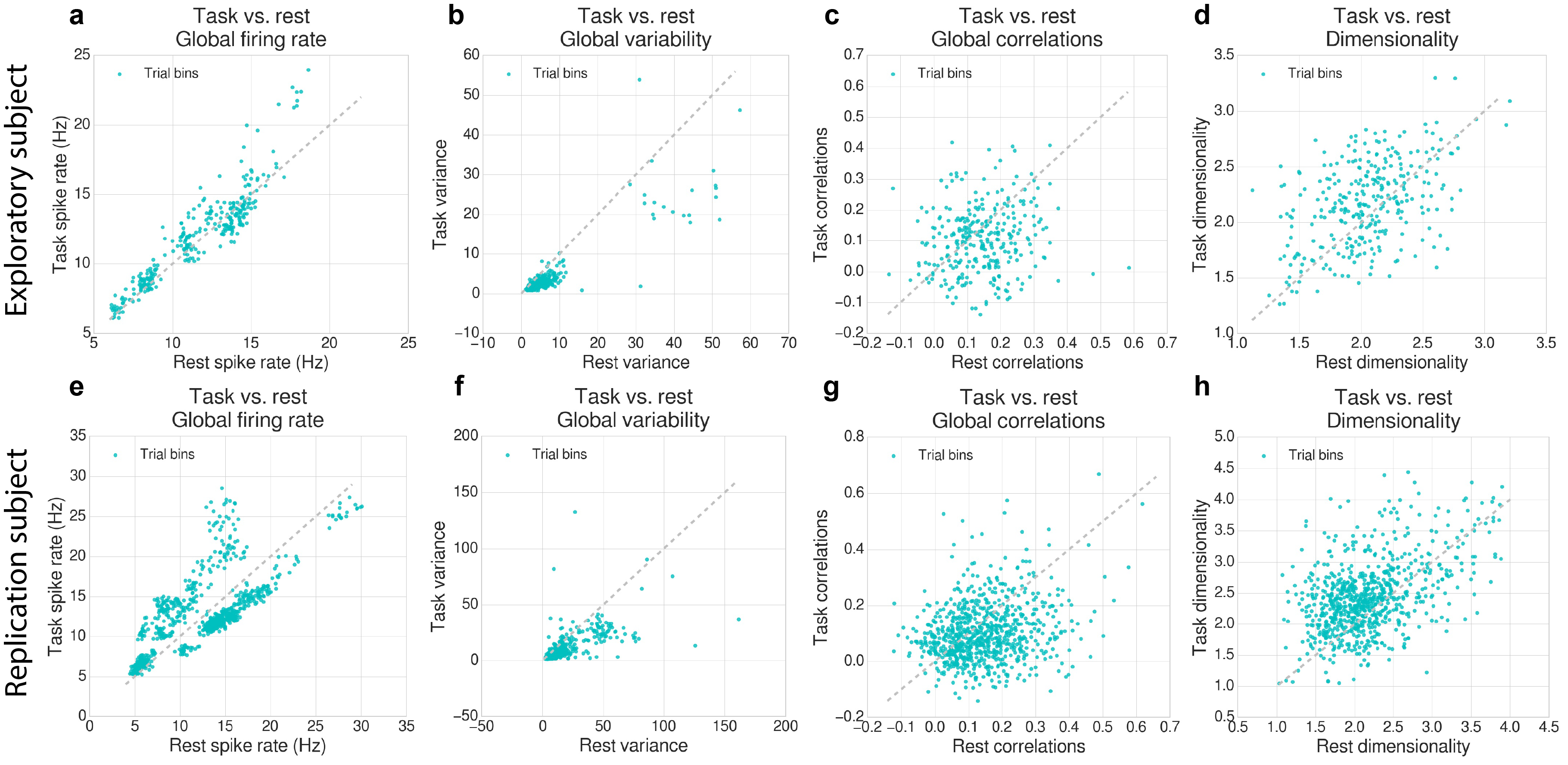

Supplement: S15 Fig — In each scatter plot, every point reflects the statistic (mean, variance, correlations, dimensionality) estimated across a bin of 25 contiguous trials. (Rest periods were defined as the ITI preceding the task cue onset.) Statistics were averaged across all recording sites, and included all recording sessions. a) The firing rate (averaged across six cortical areas) for task (y-axis) and rest (x-axis) states. b) The variance (averaged across six cortical areas) for task (y-axis) and rest (x-axis) states. c) Correlations (averaged across all pairwise correlations) for task (y-axis) and rest (x-axis) states. d) Dimensionality (i.e., participation ratio) of all six cortical areas during task (y-axis) and rest (x-axis) states. e-h) The same as a-d, but for the replication subject. (TIF) [file pcbi.1007983.s015.tif]
