# Supplementary material for: Task-evoked activity quenches neural correlations and variability across cortical areas
Source: PLoS Comput Biol. 2020 Aug 3;16(8):e1007983. doi: 10.1371/journal.pcbi.1007983 (PMC7425988; doi:10.1371/journal.pcbi.1007983)
Supplement: Attachment — Submitted filename: ResponseToReviews.pdf [file pcbi.1007983.s016.pdf]

Response to Reviews, "Task-evoked activity quenches neural correlations and variability across cortical areas"

We thank the editor and three reviewers for their evaluation of our manuscript. We are grateful for the overall positive assessment, the constructive criticisms, and the opportunity to submit a revised version of our manuscript.

We believe the feedback and subsequent revisions have significantly improved the manuscript. We have significantly clarified the manuscript text to avoid any potential conceptual and technical misunderstandings. Among other revisions, some major additions in the revised manuscript include: 1) The inclusion of a large-scale network model (300 units) to illustrate that the findings in our minimal (1 and 2 unit models) scale to larger networks (S11 Fig), 2) New visualizations (scatter plots) that describe the relationship of rest-to-task changes in variability and correlations/covariance in the human fMRI data (Fig 2, S4 Fig, S5 Fig); 3) Additional analyses demonstrating that the fano factor is reduced in our NHP data at both the mean-field (S14 Fig) and individual neuron-level (S12 and S13 Fig); 4) New scatter plot visualizations, detailing the changes to spike rate, variability, correlation, and dimensionality during ongoing and task-evoked states in the NHP data (S15 Fig).

Below, we address point-by-point criticisms raised by the three reviewers, and where appropriate, reference the specific revisions in the manuscript that address each point. (Reviewer comments in italics. Text revisions to the manuscript in yellow highlight.)

Reviewer #1: I think this is a nice paper and I recommend publication.

****

1. We thank Reviewer 1 for their positive assessment of our manuscript.

****

Reviewer #2: The authors analyse two different datasets (human fMRI and NHP electrophysiological data) for spontaneous and evoked activity under different tasks. They monitor the variability and correlations in the signal in rest and task conditions concluding that the task reduces the variability and the correlations in the signal compared to the rest state. The study is performed carefully and the authors provide evidence in support to their conclusion. They also perform numerical simulations for a minimal model to provide an interpretation of the observed reduced variability in terms of the dynamical evolution of the systems in resting conditions and under tasks.

The manuscript attacks a very intriguing problem of wide interest in the community. My criticism mainly derives from the apparently missing lack of a systematic control of the stimulation in the experimental protocol. More precisely, I am not surprised that the variability in the signal is reduced by the tasks. This is an expected result as the reduction of the fluctuations of the magnetization in a ferromagnet by the application of a large magnetic field. The strong

interaction with the field aligns all spins in the field direction.
****

2. We thank the reviewer for the overall positive assessment of our manuscript.

A primary premise of our analysis is based on the evidence that fMRI signals are indirectly related to underlying neural population activity. Several studies, including a recent study using simultaneous multi-unit activity, deconvolved wide-field calcium imaging data, and optical imaging (i.e., to capture the BOLD signal) have indicated that a significant of variance in the BOLD signal is associated with neural population activity (both LFP and spiking). Specifically, that study showed that deconvolved wide-field calcium imaging signals explained between 60-70% of the variance of a deconvolved BOLD signal (Ma et al. 2016). Older studies have also illustrated that the BOLD signal is not only related to LFP signals (r^2=0.521), but also with multi-unit activity (r^2=0.445) (Logothetis et al. 2001). One of the goals of our manuscript was to build on these studies to demonstrate that properties of neural dynamics (e.g., changes to correlation and variability structure during task states) were observable in both neural and fMRI data. However, while these inferences are limited given that fMRI imaging and electrophysiology were performed separately and in two different species, we believe that our analyses helps to bridge an important gap between two distinct subfields within neuroscience (human fMRI and NHP electrophysiology) to provide a more holistic understanding of task-dependent correlation/variability changes. However, we have mentioned the limitations of these assumptions in our results in the Discussion (p. 23):

"Despite replication of results across species and task paradigms, our conclusions are based on independently obtained data sets from two species and across task designs. Thus, to ensure variability and correlation quenching are from the same neural origin, it will be important for future work to investigate these questions with experimental designs that simultaneously record both BOLD signal and spiking activity across multiple cortical areas."

R2 also suggested that the reduction in variance is due to the field alignment in a large magnetic field during stimulation. While this may be theoretically plausible, we argued [above] and in the Discussion that neural dynamics are strongly associated with BOLD dynamics. In addition, in our numerical simulations that simulate spike rate dynamics, a nonlinear transformation of the spike rate signal to the BOLD signal (via the Balloon-Windkessel model (Friston, Harrison, and Penny 2003)) preserves variability reduction changes (SFig 6), suggesting that variability reductions would be observed independent of fluctuations induced by changes to the magnetic field. We have emphasized this distinction in the Discussion (p. 23):

"In addition, our computational model results demonstrated that reductions in neural variability and correlations were preserved after nonlinearly transforming the spike rate signal to the fMRI BOLD signal with the Balloon-Windkessel model (Friston, Harrison, and Penny 2003), suggesting that the observed signal changes are likely due to the BOLD signal changes rather than MRI artifacts."

****

The main conclusion by the authors would be more convincing if observed systematically by controlling the amplitude of the external stimulation. The task is not well defined in the manuscript and the duration of the application of some seconds suggests that the stimulation is quite large. Moreover, I think that applying, in a sequence, different stimulations (not otherwise specified) is also a non-rigorous procedure. Indeed, it is possible (as suggested by numerical simulations) that the application of a short in time small amplitude stimulation would not reduce the variability.

****

3. We apologize that the manuscript was not clearer. In our empirical data, the amplitude of the external stimulation was controlled for *by each task condition separately*. This meant that 'stimulations' (or activations) of the same condition (e.g., 2-back for faces in the working memory task) were controlled for separately from stimulations of a different condition (e.g., 2-back for tools in the working memory task). Though the precise temporal shape of the task-related stimulation for each task condition is unknown (and likely changes depending on the brain region), the external stimulation was flexibly accounted for by using finite impulse response (FIR) regression. This meant that each task condition (or, 'stimulation') was flexibly and rigorously accounted for uniquely. We have added a sentence clarifying this in the Methods section (highlighted below), detailed in the Methods section "fMRI: Preprocessing" (p. 28):

"FIR modeled task blocks were modeled separately for task conditions within each of the seven tasks. Thus, the mean task-evoked activation was differentially accounted for according to each specific task condition. In particular, two conditions were fit for the emotion cognition task, where coefficients were fit to either the face condition or shape condition. For the gambling reward task, one condition was fit to trials with the punishment condition, and the other condition was fit to trials with the reward condition. For the language task, one condition was fit for the story condition, and the other condition was fit to the math condition. For the motor task, six conditions were fit: (1) cue; (2) right hand trials; (3) left hand trials; (4) right foot trials; (5) left foot trials; (6) tongue trials. For the relational reasoning task, one condition was fit to trials when the sets of objects were matched, and the other condition was fit to trials when the objects were not matched. For the social cognition task, one condition was fit if the objects were interacting socially (theory of mind), and the other condition was fit to trials where objects were moving randomly. Lastly, for the working memory task, 8 conditions were fit: (1) 2-back body trials; (2) 2-back face trials; (3) 2-back tool trials; (4) 2-back place trials; (5) 0-back body trials; (6) 0-back face trials; (7) 0-back tool trials; (8) 0-back place trials. Since all tasks were block designs, each time point for each block was modeled separately for each task condition (i.e., FIR model), with a lag extending up to 25 TRs after task block offset."

This was also mentioned in another Methods section, "fMRI: Task state activation analysis". Note, that for task activation analysis we used a canonical HRF to model the activation

amplitude (rather than FIR model). We have added a clause (highlighted) to emphasize that activations were obtained for each condition separately (p. 28):

"We performed a task GLM analysis on fMRI task data to evaluate the task-evoked activity. The task timing for each of the 24 task conditions was convolved with the SPM canonical hemodynamic response function to obtain task-evoked activity estimates for each task condition separately [65]. FIR modeling was not used when modeling task-evoked activity. Coefficients were obtained for each parcel in the Glasser et al. (2016) cortical atlas for each of the 24 task conditions."

****

It would be also more appropriate to analyse the variability by averaging the response to the same task, not a series of different ones as I understand from the manuscript. I therefore suggest to analyse data for different durations of the stimulation applied, by using the same stimulation frame not a sequence of different ones, whose effects on the evoked activity can be averaged out in the statistical procedure.

****

4. Though for the main manuscript we analyze how variability is reduced across all task conditions together (after accounting for the condition-specific activation responses separately), we also illustrate results for variability reductions for each task separately (averaged across conditions within each task, though mean activations were removed for each condition separately within each task) (SFig 8a).

While we were unable to systematically vary stimulation strengths across different trials (e.g., via controlling for stimulation amplitude via TMS or systematic control of visual stimulation, such as contrast/luminance control), we showed that the amount of task-evoked activation (i.e., how strong a neural/fMRI response is to a given task condition) was negatively correlated with variability reduction across brain regions (S3 Fig). This suggested that the stronger the response to stimulation (i.e., task activation) in a brain region, the stronger the variability reduction was in response to that brain region. This analysis also performed for each task separately (SFig 8b).

****

Finally, drawing conclusions on data collected from two humans only is also not very rigorous.

****

5. Our human data set actually contained 176 subjects from the HCP data set, and then was replicated in a new set of 176 subjects, demonstrating the robustness of these effects across subsets of the general population (see Methods section, "fMRI: Data and paradigm").

However, our non-human primate data was only collected in two subjects. This small sample size in subjects is standard in the non-human primate electrophysiology literature, given the difficulty of training and performing surgeries on large NHP populations (e.g., see (Brincat et al. 2018; Siegel, Buschman, and Miller 2015)). Importantly, in return for the two subjects, a large number of trials was obtained in each subject, providing high-powered and statistically robust results for each subject independently. Thus, given the large number of trials for each non-human primate, we took the approach of analyzing each subject separately in our non-human primate data set, rather than performing a group-analysis. We agree with R2 that a group test across two subjects would provide statistically underpowered results.

This approach to analyzing subjects individually has recently gained popularity in several human fMRI studies, where large amounts of data are obtained for individual subjects, rather than small amounts of data across a large subject cohort (Huth et al. 2012; Nakai and Nishimoto 2020; Gordon et al. 2017). In this approach, each additional subject in the data set serves as replication to the initial subject (since each subject is analyzed separately).

****

As a minor remark, the author should specify what they mean by a negative stimulation amplitude in the numerical study and how this would compare to experimental data. Moreover, since the authors relate the reduced variability to the high activity level induced by the task, how can they justify the reduced variability by a de-activation stimulation?

****

6. In the numerical simulation, negative stimulation is indicated by a negative input value into the neural mass. In the one and two-dimensional firing rate models (governed by equations 8 and 10/11, respectively),

$$\tau_i \frac{dx_i}{dt} = -x_i + f(w_{ii}x_i + b_i + s_i + I) \quad (8)$$

$$\tau_1 \frac{dx_1}{dt} = -x_1 + f(w_{11}x_1 + w_{21}x_2 + b_1 + s_1 + I_1) \quad (10)$$

$$\tau_2 \frac{dx_2}{dt} = -x_2 + f(w_{22}x_2 + w_{12}x_1 + b_2 + s_2 + I_2) \quad (11)$$

A negative stimulation refers to a negative value for the s_i parameter (p. 32). In our spiking model (Fig. 6a,b), this corresponds biophysically to the activation of the inhibitory neurons within a local population. We have clarified the phrasing in the Methods sections, “Model: One-dimensional minimal network model” and “Model: Two-dimensional minimal network model” to reference how s_i is defined:

In “Model: One-dimensional minimal network model”, p. 32:

“We simulated neural population activity injecting a fixed input (boxcar input) with amplitudes ranging from $s_i \in [-5, 5]$ in 0.01 increments (Fig 7C).”

and

“Each trial was run for 20 seconds. Fig 7C was generated using input amplitudes of $s_i \in \{-3, 0, 3\}$.”

In “Model: Two-dimensional minimal network model”, p. 33:

“We injected fixed input into both neural populations with amplitudes ranging from $s_i \in [-5, 5]$ in 0.01 increments (Fig. 7E)”

We apologize to the reviewer for not making our being clearer; the results portion that indicates that more task-active regions reduced their variability was poorly phrased. The results illustrated an association of task-evoked activation *magnitude* with variability reduction (S3 Fig). (Magnitude here reflects the absolute value of the task-evoked activation coefficient, which is estimated from a task GLM. This indicates that the magnitude of either positive *or negative* activation coefficients is associated with variability reductions. This was more precisely indicated in the S3 Fig axis labels.) This analysis captures the magnitude of fMRI BOLD deflections relative to baseline, independent of the sign of the deflection. This analysis was motivated by and replicates a previous study that showed that voxels with a greater fMRI BOLD activation magnitude had greater variability reductions (see Figure 4A in (He 2013)). To better characterize our results, we have rephrased our wording to indicate that task-evoked activation *magnitudes* are correlated with variability and correlation reductions. Critically, this analysis is consistent with our model, which suggests that highly active *or deactive* states would result in variability reductions. The results paragraph has been revised as follows (see p. 12):

“Previous work has shown that regions that have strong task activations (i.e., the magnitude of the task-induced activation, positive or negative) tend to have greater variability reductions (He 2013). (Task activation magnitudes reflect the deviation of the BOLD activity relative to baseline, or the inter-block interval.) We sought to replicate this effect in the current data set, while extending those results to demonstrate that more task-active regions also tend to reduce their FC during task states. We first correlated regional task-evoked activation magnitude with task-evoked variability reduction (task variance minus rest variance) across regions at the group-level. We found that regions with greater task-evoked activation magnitudes (averaged across tasks) exhibited greater variability reductions during task states, confirming previous findings in a finger tapping task (exploratory cohort rho=-0.32, p<10e-9; replication cohort rho=-0.49, p<10e-22; Supplementary Fig 3A,C) (He 2013). This negative relationship was also observed in 6/7 of the HCP tasks when analyzed separately (FDR-corrected p<0.01; S8 Fig). To link regional task activation magnitudes with FC decreases, we tested for a correlation between regional task-evoked activation magnitude and the average FC change during task states for each region. Consistent with our hypothesis, we found that regions with greater task-evoked activation magnitudes (averaged across tasks) reduced their average FC more during task states (exploratory cohort rho=-0.25, p<10e-05; replication cohort rho=-0.20, p=0.0002; S3 Fig). When tasks were analyzed separately, this negative correlation was observed in 4/7 of the HCP tasks (FDR-corrected p<0.05; S9 Fig). Thus, brain areas with higher levels of task-evoked activation magnitudes (i.e., changes in activity relative to baseline) tend to reduce both their task-evoked variability and FC.”

****

Reviewer #3: In behaving animals, pairwise correlation among neurons in a local network is generally reduced during evoked activity. By contrast, at larger-scale when we study interactions across brain regions we find that correlations (functional connectivity: FC) can both increase and decrease. Authors claim that taken together these observations are incompatible. In this study authors have addressed this problem. To this end, they have analysed synchrony and variability in spiking activity recorded from primates and in fMRI signals recorded from humans. They argue that unlike previous observations, task related activity quenches correlations and variability across cortical areas (fMRI measurements). They provide an explanation of reduction in cortical variability and correlation in evoked activity in terms of attractor dynamics. The key to the explanation is neuron mass model transfer function and how the attractor is shifted by the inputs.

Overall there is huge amount of work has gone into this manuscript: from analysis of spiking activity and fMRI data to simulation of neuron mass models with one and two populations. But I think manuscript needs some more work in terms of data analysis to really establish the results based on the experimental data. The major problem with the manuscript is the choice of the model which is not suitable to address the problem. Moreover, the even though the explanation based on the results seems fine it does not apply to the neuronal networks in the brain. So we have an experimental observation at the level of inter brain region correlations not only that needs to demonstrated with greater rigour but also there is no explanation of the observations. In the following I elaborate on these.

****

7. We thank Reviewer 3 for a thorough evaluation of our manuscript. Below we address the critiques in more detail and highlight corresponding revisions.

****

Major:

- At the outset I do not agree that there is any incompatibility in reduction in spiking activity correlations and increase/decrease in fMRI-based FC. There is no reason why these two should be similar. Spiking activity correlation reduction is a property of the local network dynamics and FC is about inter-region correlations. Not only that these correlations are measured at different time scales but the local and inter-brain region connectivity are very different.

- I agree that here authors have analysed spike level correlations using multi-unit activity between different brain regions and found that there is a reduction in both variability and correlations. Even though authors suggest that they have used consistent statistical methods to analyse spikes and fMRI signals, the underlying signals have completely different origins and

are measured at very different time and spatial scales. So again, I do not think there is any reason to believe that modulation of correlation should be similar in multi-unit and fMRI signals.

****

We sincerely apologize for not being clearer - it appears that there was a core misunderstanding of how we analyzed the NHP data.

8. Addressing the issue of incomparable spatial resolutions:

While multi-unit spiking data was collected for the NHP data set, we did not use the individual units for analyses. Instead, we computed the mean-field spike rate within each of the six cortical areas. Though this is an unusual approach for multi-unit spiking data sets because information and spatial specificity is thrown out due to spatial downsampling, we intentionally chose this approach to better match the spatial resolution of our fMRI data (i.e., mean-field cortical areas, rather than individual neurons). For this study, we were interested in making statistical inferences at the level of large neural populations (i.e., inter-area dynamics), rather than at the local network (Doiron et al. 2016). We acknowledge that the approach of averaging spikes across the multi-units prevents us from making any inferences at the level of a local population.

Importantly, computing the mean-field spike rate of a cortical area (by averaging multi-units) provides a better spatial comparison to our fMRI data. Moreover, few NHP spiking data sets are well-positioned to investigate inter-region correlations for more than two regions given the overall difficulty of obtaining simultaneous electrophysiological recordings from multiple cortical sites. Thus, while we significantly simplified (downsampled) our spatial recordings, we leveraged this unique NHP data set to directly address mean-field, inter-region correlations to facilitate translation to our human fMRI data. More broadly, we sought to bridge the gap between human neuroimaging and NHP electrophysiology (particularly with respect to “functional connectivity” and “noise correlation” analyses), since few links between these subfields are often made.

R2 raised related doubts regarding whether the neural origins of fMRI and mean-field spiking activity are related. We believe the work of Nikos Logothetis’ group and more recently, Elizabeth Hillman’s group, have provided strong evidence that the fMRI signal has neural (spiking) origins (Logothetis et al., 2001; Ma et al., 2016). We have addressed these concerns to R2 (see response #2 above), and have revised the manuscript accordingly. In general, we have substituted the use of ‘multi-unit’ with ‘mean-field’ (with the exception of the Methods section, where we detail that mean-field spike rates were derived from the average spiking activity of a multi-unit recording):

“First, we characterize task-evoked neural variability and correlations in empirical data using two highly distinct data sets: multi-site and mean-field NHP spike rates and whole-brain human fMRI… The combination of simultaneously recorded mean-field spike rate recordings from six cortical sites, whole-brain fMRI obtained from seven different cognitive tasks, and dynamical

systems modeling and analysis provide a comprehensive account of task-related correlation and variability changes spanning species and data acquisition techniques.” (p. 3, Introduction)

“NHP mean-field spiking and human fMRI data… In particular, we focused on neural variability and correlation changes across large cortical areas (mean-field) in our electrophysiology data set (rather than between pairs of neurons) given our focus on large-scale neural interactions, and to facilitate a comparison between different correlation approaches (FC in fMRI data and spike count correlation in electrophysiology data).” (p. 4, Results)

“**a,b)** Using mean-field spike rate data collected simultaneously from six different cortical areas (Siegel, Buschman, and Miller 2015), we compared the spiking variability and spike count correlations between task-state (i.e., following task cue onset) and rest-state spiking activity… This was performed by estimating the mean-field spike rate by averaging across multi-units in each cortical area, allowing us to target the activity of large neural populations.” (p. 5, Fig 1 caption).

“We measured mean-field spike recordings from six different cortical areas during a motion-color categorization task.” (p. 6, Fig 2 caption)

“In the previous sections, we provided empirical evidence that task states reduce mean-field inter-area correlations and variability in spike rate and fMRI data.” (p. 14)

“**a)** Mean-field spike recordings from six different cortical regions fed into our analyses.” (p. 41, S1 Fig caption)

9. Addressing the issue of incomparable temporal resolutions:

R3 also raises an important point regarding the fact that fMRI data and spiking data are sampled at different timescales. To mitigate this issue, variability and correlations for our NHP data set were computed *across trials*, where spiking activity was temporally downsampled (to obtain spike counts per trial). This placed the temporal time scale of our NHP data (500ms-1000ms per trial) in line with our fMRI data (sampled at 720ms), such that the temporal timescales were on similar orders of magnitude. We have added a clarifying sentence in the beginning of our Results section to highlight our effort to match the temporal resolution of spiking and fMRI data:

“In addition to spatially downsampling our NHP data to evaluate mean-field spike rates in each cortical area, we also temporally downsampled our NHP data to investigate variability and correlation changes across trials (on the order of hundreds of milliseconds), which appropriately matches the sampling rate of our fMRI data (720ms).” (p. 4, Results)

In addition, in our numerical simulations, we verified that reductions in variability and correlations were preserved after nonlinearly transforming the original spike rate signal to fMRI signal (SFig 6). This analysis showed that similar changes to neural dynamics (i.e., variability/correlation changes) are likely to be observed despite the application of a low-pass

filter to high temporal resolution data (i.e., neural-to-BOLD signal transformation). This was highlighted in a paragraph in the Discussion section:

“Despite converging results, there are several key differences in our two empirical data sets. First, the time scale of fMRI BOLD activity is much slower than the NHP spiking activity. However, these differences were mitigated by measuring spiking variability across trials, which is comparable to the time scale of fMRI’s sampling rate (in the hundreds of milliseconds). In addition, our computational model results demonstrated that reductions in neural variability and correlations were preserved after nonlinearly transforming the spike rate signal to the fMRI BOLD signal with the Balloon-Windkessel model [41].” (p. 23, Discussion)

****

- I do not think that showing just the variance of activity across trials is a proper measure of the variability. In network models when neurons are connected it may be possible that the mean and variance of the activity are inter-related. But the real reason for measuring Fanp Factor (FF) is that we want to compare with an unbiased uncorrelated population of homogeneous Poisson type spike train ensemble. Change in the variance between

resting and evoked activity state could simply be because of a change in the mean responses. So I insist on showing FF as a measure of the variability.

****

10. We have re-analyzed our data and calculated the fano factor for NHP data. Results are reported in S14 Fig for global (averaged across all sites) fano factor, and S12-13 Fig for the fano factor of each neuron within each of the six cortical sites. Note that our original analyses/inferences were limited to mean-field analyses, but we reported FF statistics for individual neurons (across all recording sites/sessions) for completeness and future reference. We have included text in the Results reporting that results are unchanged when using FF, and that at the individual neuron level, a majority of neurons decrease their FF during task states (p. 6):

“Variability reductions were also observed using fano factor (rather than variance) at both the mean-field (averaged across neurons; S14 Fig) and for the majority of individual neurons in each cortical area (S12 and S13 Fig).”

S14 Fig (p. 58):

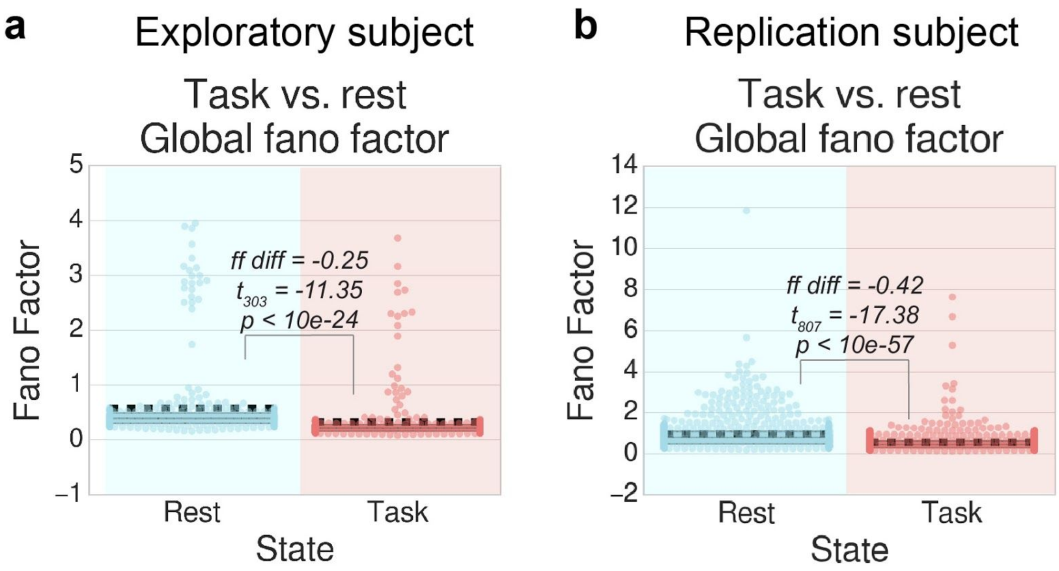

S14 Fig. The average fano factor change from rest (ITI) to task (cue) periods for both the exploratory and replication NHP subjects. a) Exploratory subject. **b)** Replication subject.

S12 Fig, the exploratory subject (p. 56):

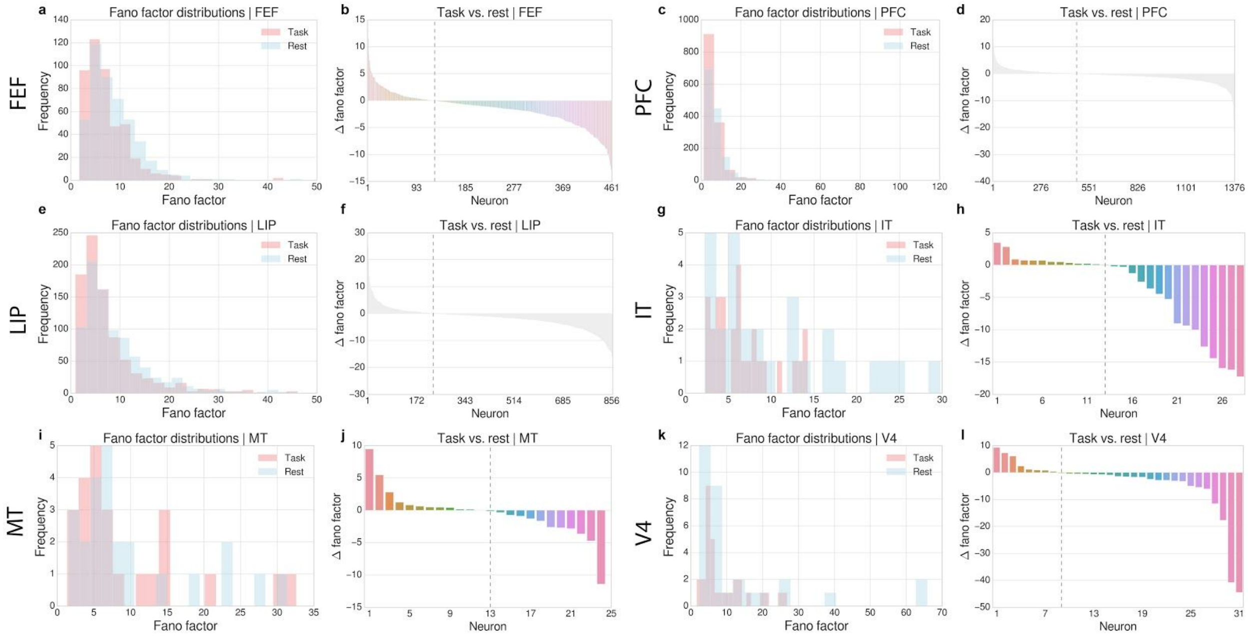

S12 Fig. Rest (ITI) to task state (task cue) changes in fano factor analyzed for each neuron individually across the six cortical areas for the exploratory subject. (This is *not* a mean-field analysis.) **a)** The distribution of fano factor across all neurons in FEF (from all recording sessions) for the rest (ITI) and task state (cue) periods. **b)** For each individual neuron in FEF, we calculated the change in fano factor from the rest to task state period. **c,d)** Same as **a, b**, but for PFC. **e,f)** Same as **a, b**, but for LIP. **g,h)** Same as **a, b**, but for IT. **i,j)** Same as **a, b**, but for MT. **k,l)** Same as **a, b**, but for V4.

S13 Fig, the replication subject (p. 57):

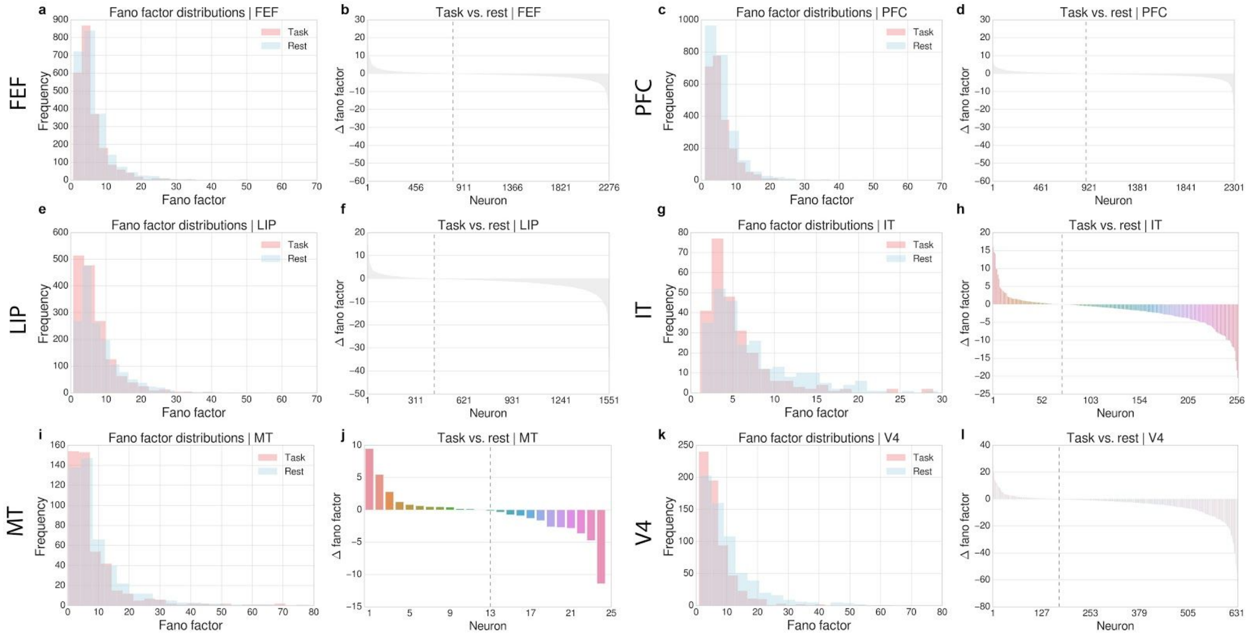

S13 Fig. Rest (ITI) to task state (task cue) changes in fano factor analyzed for each neuron individually across the six cortical areas for the replication subject. (This is *not* a mean-field analysis.) **a)** The distribution of fano factor across all neurons in FEF (from all recording sessions) for the rest (ITI) and task state (cue) periods. **b)** For each individual neuron in FEF, we calculated the change in fano factor from the rest to task state period. **c,d)** Same as **a, b**, but for PFC. **e,f)** Same as **a, b**, but for LIP. **g,h)** Same as **a, b**, but for IT. **i,j)** Same as **a, b**, but for MT. **k,l)** Same as **a, b**, but for V4.

****

- An additional confound is that here authors are using multi-unit spike trains. The variance of multi-unit spike train can also change if the correlation between single-units (which constitute the multi-unit) changes (even if weakly) -- variance of multi-unit spike train is sum of the variance and co-variance of single units that constitute the multi-unit. So if different populations of neurons are active in evoked and task related state, the comparison of variance becomes very tricky.

****

11. We thank the reviewer for this possible confound. We agree that it is possible that different populations of neurons are active during spontaneous versus task-evoked states. However, because we used mean-field spike rates (rather than multi-unit spikes), the discrepancy of different populations of neurons (within the same cortical area) is lost. Though the possibility of task-active versus inactive population activity driving differences in mean-field neural spiking statistics is interesting, we believe that this is beyond the scope of this manuscript, given that such a comparable analysis would be difficult (if not impossible) in our fMRI data.

****

- The difference in evoked and ongoing spike rates are very small? Why is it so?

****

12. During the process of re-analyzing our data to perform fano factor analyses, we realized that we had incorrectly converted spikes to Hz. (Instead of multiplying by a factor, we divided by this factor). **Thus, we have re-analyzed and updated all figures/statistics with this correction. This includes Fig 2, Fig 5B, S1 Fig, S2 Fig. Importantly, no fundamental results were altered by this,** except for a change in observed covariance increase that survived statistical thresholding (this point is discussed below)**.**

In general, the ongoing and evoked spike rates (averaged across all recording sites) range from 10-15Hz (Fig 2, p. 6):

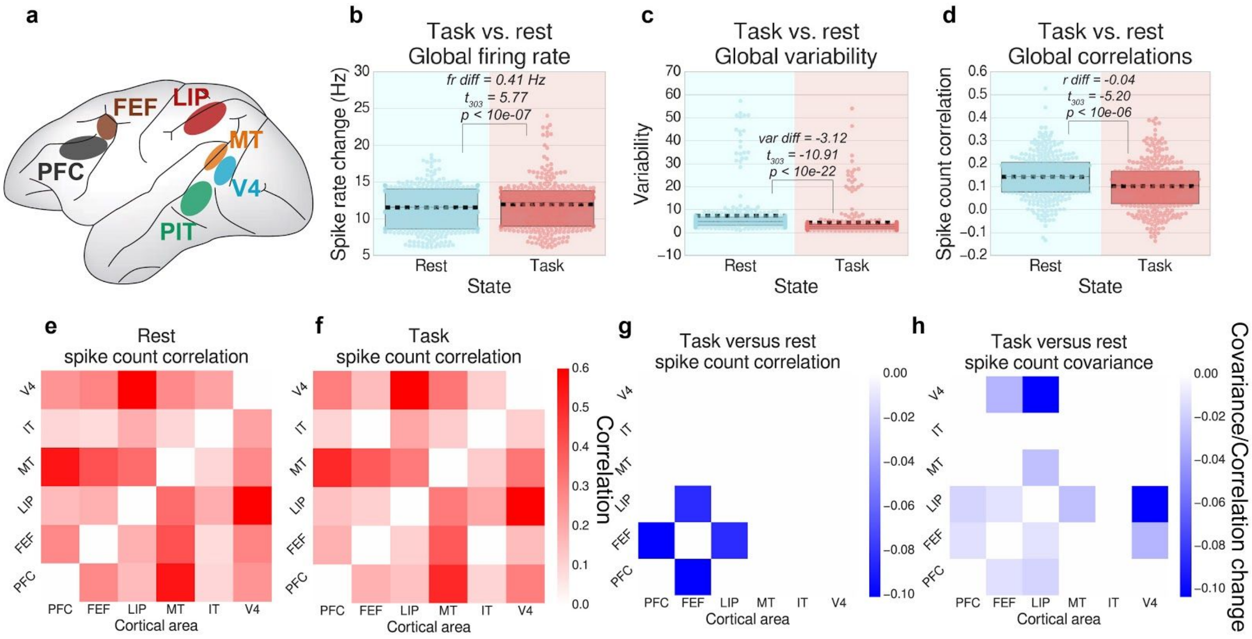

Fig 2. Neural variability and correlations decrease during task states relative to rest in spiking data. Results for the replication subject are reported in S1 Fig. **a)** We measured mean-field spike recordings from six different cortical areas during a motion-color categorization task. **b)** We calculated the average spike rate change across all recordings during the rest period (ITI) and task period (task cue), across trials. Each data point reflects the firing rate across 25 consecutive trials. **c)** We calculated the cross-trial spiking variance for each region during task and rest states, and then averaged across all regions. Each data point reflects the spiking variance across 25 consecutive trials. **d)** We calculated the average cross-trial neural correlation for task and rest states between all pairs of recorded brain regions. (Spike rates were averaged within each cortical area.) Each data point reflects the correlation across 25 consecutive trials. **e-g)** For each pair of brain regions, we visualize the correlation matrices between each recording site for the averaged rest period, task period, and the differences between task versus rest state spike count correlations. **h)** We also observed no increases in covariance (non-normalized correlation) (Duff et al. 2018; M. W. Cole, Yang, et al. 2016; Siegel, Donner, and Engel 2012). For panels **e-h**, plots were thresholded and tested for multiple comparisons using an FDR-corrected $p<0.05$

threshold. Boxplots indicate the interquartile range of the distribution, dotted black line indicates the mean, grey line indicates the median, and the distribution is visualized using a swarm plot.

****

- Authors argue that in task-state correlation increase in the replication subject is because of a decrease in unshared variance (Page 7 first para). I am not sure I understand the line of argumentation. Could authors format this in mathematical equations.

****

13. After re-analysis of our data with the corrected conversion to Hz mentioned above, we found that the previously reported increases in correlation in the replication NHP were reproduced using measures of covariance (un-normalized correlation). (Note, however, that we did not find any correlation/covariance increases in the exploratory NHP.) Though the overall increase in covariance was fairly weak, these results did not change the primary finding that the majority of pairwise correlations (and covariances) were strongly reduced. Interestingly, the correlation/covariances that increased were between visual areas and frontal areas (i.e., MT and IT with PFC and FEF). While these mean-field spike count correlation increases have not been reported in the literature, these increases appear to be consistent with our fMRI data (which see correlation increases among the frontoparietal network and the visual network; see Fig 3). Though these mean-field correlation increases between frontal and visual areas appear to be a novel finding in the NHP electrophysiology literature, we believe a full analysis on these correlation/covariance increases are beyond the scope of this manuscript. Moreover, these correlation increases were not replicated in the exploratory NHP data. Nevertheless, we hope to explore these phenomena in more detail in a future study. We have also revised the appropriate Results and Discussion sections to reference these findings.

Results (p. 7):

"We did not identify any pairwise correlation and covariance increases in our exploratory NHP (Fig 2G). However, in our replication NHP we found correlation increases between visual and frontal areas (i.e., MT/IT and PFC/FEF) (S1 Fig). When analyzed with covariance (rather than correlation), we found these covariance increases to be weak relative to the observed covariance decreases. (Moreover, the baseline correlation strength between these areas was very low during the ITI period.) Though these correlation increases were only observed in 1 of 2 NHPs, they were generally consistent with our fMRI data (below), which showed that though there were few correlation increases, variability and correlations across cortex were dominated by decreases during task states."

Discussion (p. 21-22):
"Though we largely focused on FC decreases during task states in both data sets, we identified a small number of correlations that increased during task state. Most of these correlation increases were primarily between regions belonging to different functional networks, such as frontal and visual areas, which is consistent with previous literature (M. Cole et al. 2014;

Krienen, Yeo, and Buckner 2014; Gonzalez-Castillo and Bandettini 2017). Some correlation increases have also been reported in the NHP spiking literature, where spike count correlations between units with similar task receptive fields tend to decrease, while spike count correlations between units with dissimilar task receptive fields increase (Ruff and Cohen 2014). This appears to be conceptually consistent with the present findings, where we focus on mean-field correlation changes between functionally distinct cortical areas (rather than between pairs of individual neurons). Specifically, we found that regions in the same network tend to decrease their correlations, while regions across functionally distinct areas/networks marginally increase their correlations. In our fMRI data, we found marginal task-state correlation increases between different networks, such as the frontoparietal and visual networks (Fig 3F). Correlation increases were also observed in one of the two NHPs between frontal (PFC and FEF) and visual (MT and IT) areas (S1 Fig). (However, we note we did not observe any correlation increases in our exploratory NHP.) While these correlation increases appear to be statistically reliable in the second NHP, the spontaneous (resting-state) correlations between these areas were very low and the correlation increases small. Thus, it will be important for future investigations to directly evaluate the functional relevance of these marginal correlation increases"

Re-analysis of the replication subject with corrected spiking units (S1 Fig, p. 42).

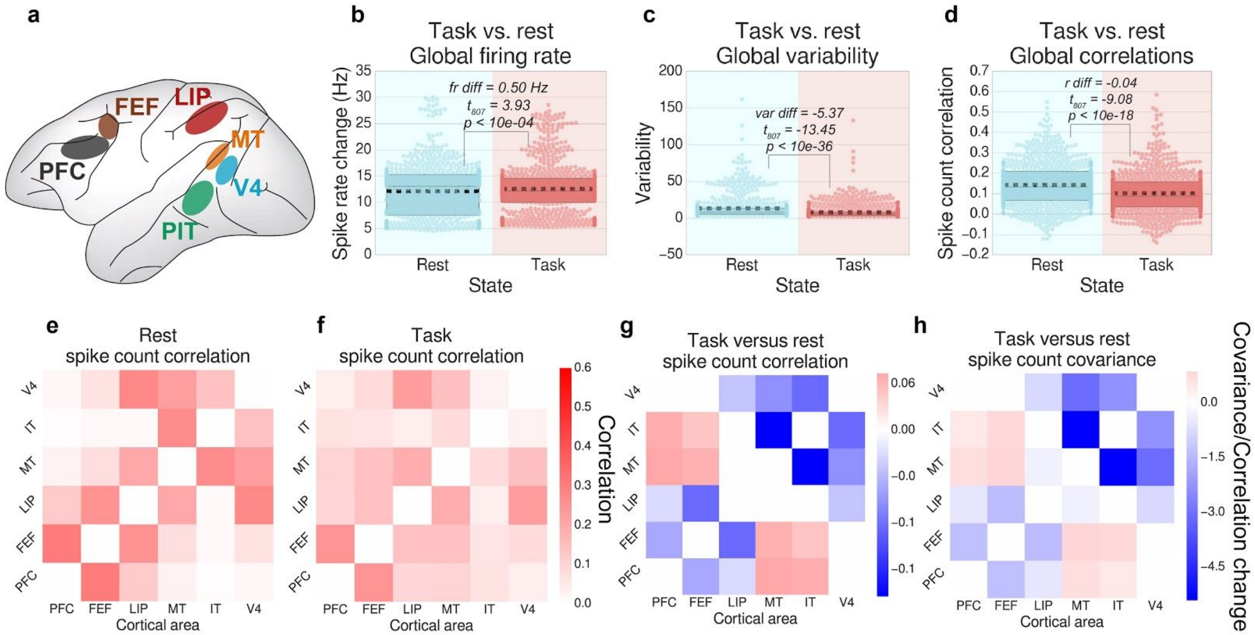

S1 Fig. Replication analysis for the excluded NHP subject. This figure is organized identically to Fig 2, but using data from a replication subject. We find nearly identical patterns between the exploratory and replication subjects, with the exception that we did not replicate any correlation increases. **a)** Mean-field spike recordings from six different cortical regions fed into our analyses. **b)** As in our empirical fMRI data set, we calculated the global variability across task and rest states (estimated using the standard deviation across trials). **c)** We then calculated the global neural correlation (i.e., the spike count correlation across trials) for task and rest states between all pairs of recorded brain regions. (Spike rates were averaged within each cortical area.) **d-f)** For each pair of brain regions, we visualized the correlation matrices between each recording site for the averaged rest, task, and the differences between task

versus rest state spike count correlations. For panels **d-f**, plots were thresholded and tested for multiple comparisons using an FDR-corrected p<0.05 threshold. Boxplots indicate the interquartile range of the distribution, dotted black line indicates the mean, grey line indicates the median, and the distribution is visualized using a swarm plot.

****

- In the section 'Task-state FC ...' authors write that 'Our current finding illustrate that mean-field spike count correlation decrease...'. Are they referring multi-unit spiking activity as mean-field activity -- if so this is wrong.

****

14. We thank the reviewer for pointing out the confusing references to both ‘multi-unit spiking activity’ and ‘mean-field spiking activity’. While multi-unit spiking activity was *recorded*, only mean-field spiking activity was *analyzed* (by averaging across multi-unit spikes). We use the following definition: “Mean Field Theory: An analysis that ignores correlations between variables so that the effect of fluctuations can be “smoothed out” by averaging and the system’s behavior described according to the mean effect of all variables that comprise a unit” (Miller 2018).

We have clarified and revised the manuscript in several parts (see response #8) to clarify that we have used the mean-field spiking activity for each recording site.

****

- In the same section 'Task-state FC ...' authors hypothesize that FC would be globally reduced. What is the basis of this hypothesis? A priori there is no reason to assume that just because correlations in local networks are reduced that the correlation between two different areas (or the FC) should also be reduced. As said earlier, fMRI FC and spike correlation are measured at very different temporal scales and most likely have different origin.

****

15. The hypothesis surrounding the fMRI task-state FC was developed based on our finding that mean-field, inter-area spike count correlations were found to be reduced in our empirical NHP data. Given the evidence that the fMRI FC is associated with synchronized activity of excitatory populations (Ma et al. 2016), we hypothesized that the inter-area correlation reductions we saw in our NHP data might extend to our fMRI data.

In responses #8 and #9 we addressed that that the cross-trial correlations in NHP data and fMRI data are sampled at comparable time scales (i.e., hundreds of ms).

****

- In Figure 3g authors show that FC is reduced in task evoked state. But this is average FC. Figure 3H is really uninformative. I do not understand what is the purpose of showing that FC of each brain area is on average reduced. If authors want to show that FC is reduced then they need to show that FC for each pair of brain regions is decreased in evoked state as compared to the ongoing activty. So I suggest that authors show a scatter diagram with x-axis as the FC of a pair in ongoing state and y-axis as FC of the same pair in evoked state. Panel 3i is good but please do not make a binary decision and show the full histogram of pair-wise difference in FC in the two states.

****

16. We thank R3 for their feedback. We have opted to keep Fig 3H, given that graph-theoretic measures, such as weighted degree centrality (or “global brain connectivity”) are commonly used network metrics that describe FC (and FC changes). Moreover, this provides a reasonable simplification of visualizing the differential effects of FC changes across a cortical surface, which is a more intuitive visualization. This contrasts Fig 3F, which shows the entire task vs. rest FC matrix (with no reference to cortical organization). However, we have taken R3’s feedback regarding Panel 3I. We have replaced it (i.e., a binary decision on FC increases v. decreases, and instead have plotted the suggested scatter plot (task-state FC v. rest-state FC for all pairs of connections). We have additionally plotted changes to regional variance as a scatter plot (Fig 3C), replacing the previous bar plot that indicated the percentage of increases/decreases in regional variance changes from rest to task. The figures (and captions) have also been updated for the replication cohort (S4 Fig), as well as the data for un-normalized variance/covariance measures (S5 Fig).

Fig 3 - note updated panels in Fig 3C and Fig 3I (p. 8).

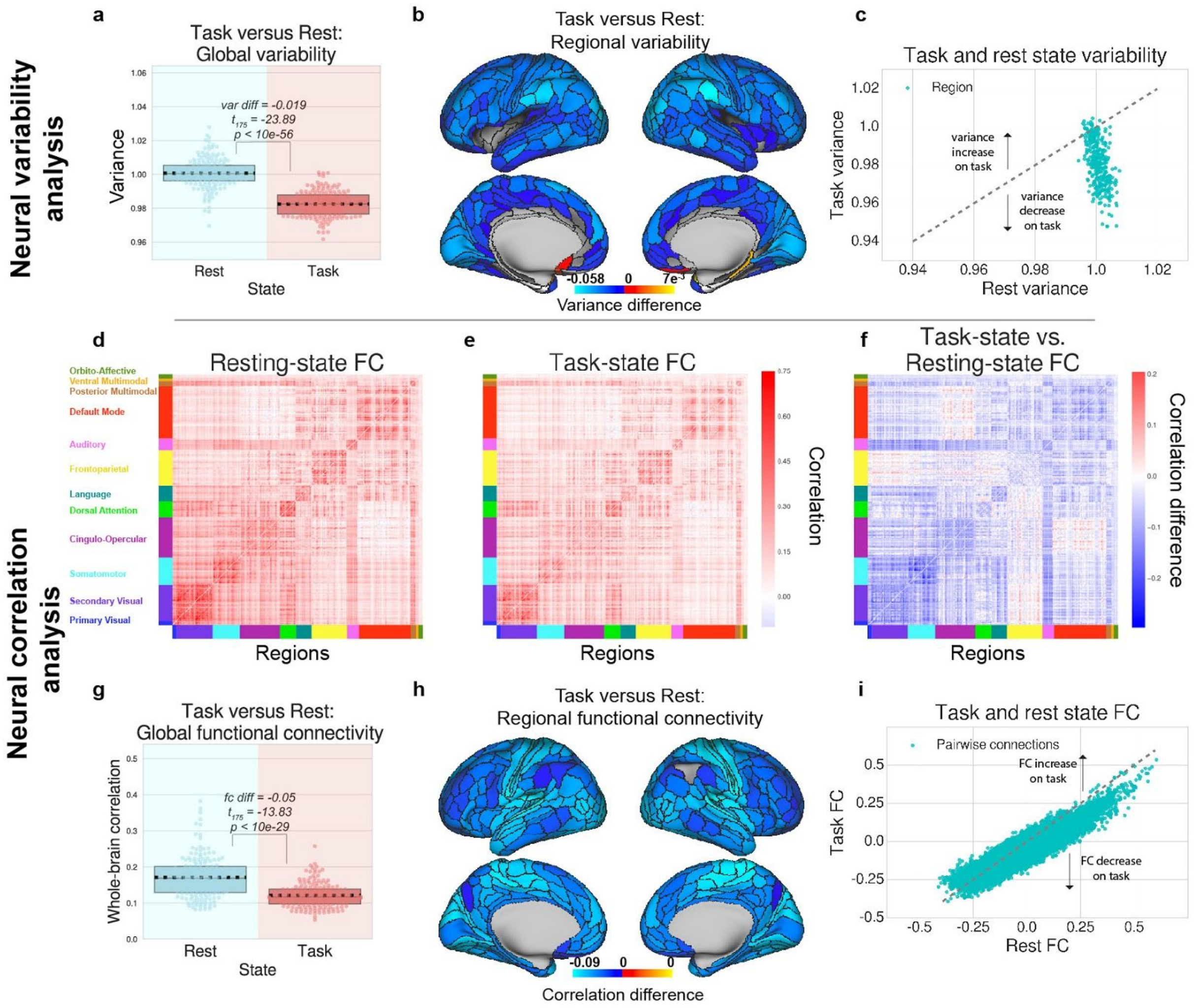

Fig 3. Variability and correlations decrease during task states in human fMRI data. Figures for the replication cohort are in S4 Fig. Figures for each task separately are shown in S8-9 Fig. **a)** We first compared the global variability during task and rest states, which is averaged across all brain regions, and then **b)** computed the task- versus rest-state variability for each brain region. **c)** Scatter plot depicting the variance of each parcel during task states (y-axis) and rest states (x-axis). Dotted grey line denotes no change between rest and task states. **d)** We next compared the correlation matrices for resting state blocks with **(e)** task state blocks, and **(f)** computed the task- versus rest-state correlation matrix difference. **g)** We found that the average FC between all pairs of brain regions is significantly reduced during task state. **h)** We found that the average correlation for each brain region, decreased for each brain region during task state. **i)** Scatter plot depicting the FC (correlation values) of each pair of parcels during task states (y-axis) and rest states (x-axis). Dotted grey line denotes no change between rest and task states. For panels **b-f,** and **h,** plots were tested for multiple comparisons using an FDR-corrected *p*<0.05 threshold. Boxplots indicate the interquartile range of the distribution, dotted black line indicates the mean, grey line indicates the median, and the distribution is visualized using a swarm plot.

Also updated figure (using un-normalized time series with variance and covariance) (S5 Fig, p. 47). Updated panels are S5C and S5I Fig:

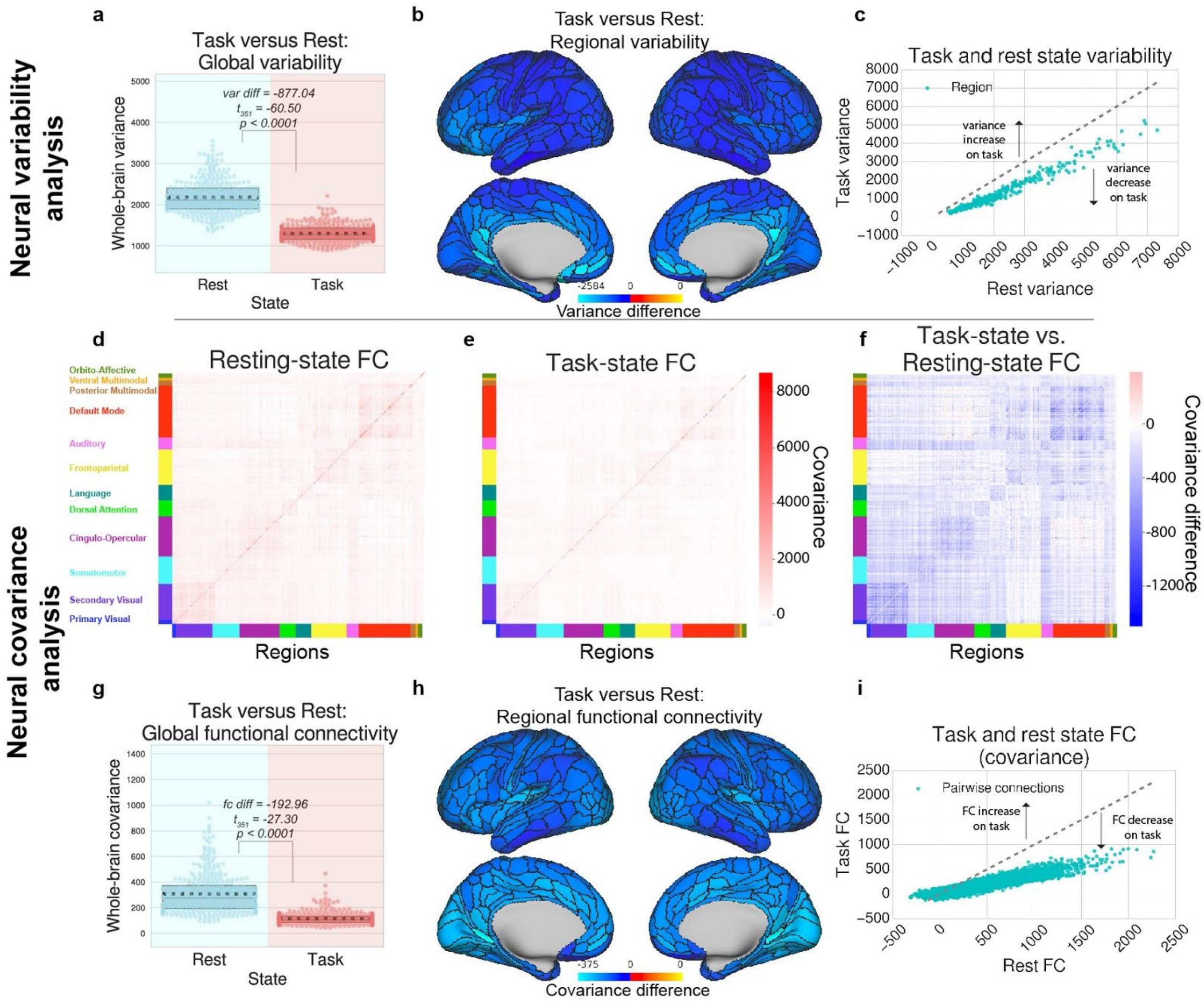

S5 Fig. Non-normalized data using variance and covariance, using the full set of 352 subjects. Neural variance and covariance decreased during task states in human fMRI data. We successfully replicated results from Fig 3 using, but without z-normalizing the time series (and using covariance instead of correlation). The combination of reduced correlations (Fig 3) and covariance measures suggested that shared signal dynamics is reduced from task to rest (M. W. Cole, Yang, et al. 2016; Duff et al. 2018; Siegel, Donner, and Engel 2012). **a)** We first compared the global variability during task and rest states, which is averaged across all brain regions, and then **b)** computed the task- versus rest-state variability for each brain region. **c)** Scatter plot depicting the variance of each parcel during task states (y-axis) and rest states (x-axis). Dotted grey line denotes no change between rest and task states. **d)** We next compared the covariance matrices for resting state blocks with **(e)** task state blocks, and **(f)** computed the task- versus rest-state covariance matrix difference. **g)** We found that the average covariance between all pairs of brain regions is significantly reduced during task state. **h)** We found that the average covariance for each brain region, decreased for each brain region during task state. **i)** Scatter plot depicting the FC (covariance values) of each pair of parcels during task states (y-axis) and rest states (x-axis). Dotted grey line denotes no change between rest and task states. For panels **b-f,** and **h**, plots were tested for multiple comparisons using an FDR-corrected p<0.05 threshold. Boxplots indicate the interquartile range of the distribution, dotted black line indicates the mean, grey line indicates the median, and the distribution is visualized using a swarm plot.

****

- Authors write that inter-area cortical network rely on excitatory connectivity -- this is correct but this does not mean that excitatory input impinges on excitatory neurons only. Long range connections impinge on both excitatory and inhibitory neurons.

****

17. We agree with R3 that long-range excitatory connectivity can have a net inhibitory effect, if the excitatory projections target the downstream local inhibitory neurons. Though our neural mass model did not incorporate this scenario explicitly, this was modeled in the balanced spiking model. Specifically, Fig. 6A-C modeled the scenario in which incoming signals targeted excitatory neurons (causing an excitatory drive locally), ***and*** the scenario in which incoming signals targeted inhibitory neurons (causing a net inhibitory effect).

We have made the corresponding revisions in the text to clarify this intuition (p. 14):

"Using this previously established model, we systematically perturbed this balanced network under a distribution of inputs (both excitatory and/or inhibitory inputs) to estimate the excitatory output (i.e., mean-field transfer function) of a cortical population. Though most long-range cortical connections are excitatory, we incorporated excitatory and inhibitory stimulation effects on a local population (Fig 6B). This is because long-range excitatory afferents may target local inhibitory neurons, producing a net inhibitory effect. Under the presence of inputs, we found that the population transfer function approximated a sigmoid activation function (Fig 6B). We note that the upper bound on the sigmoid transfer function (Fig 6D) is likely due to inhibitory feedback on excitatory activity rather than the true saturating spiking regime in neurons. This is because excitatory neurons in a local population typically do not reach a saturating spiking regime even for strong visual stimuli (Priebe and Ferster 2008), and instead reach an upper bound due to strong inhibitory stabilization preventing runaway excitation (Hennequin et al. 2018). Importantly, simplifying the mean-field transfer function of a cortical area allowed us to focus our modeling efforts on simplified networks across large cortical areas (Joglekar et al. 2018). "

Figure 6, p.15:

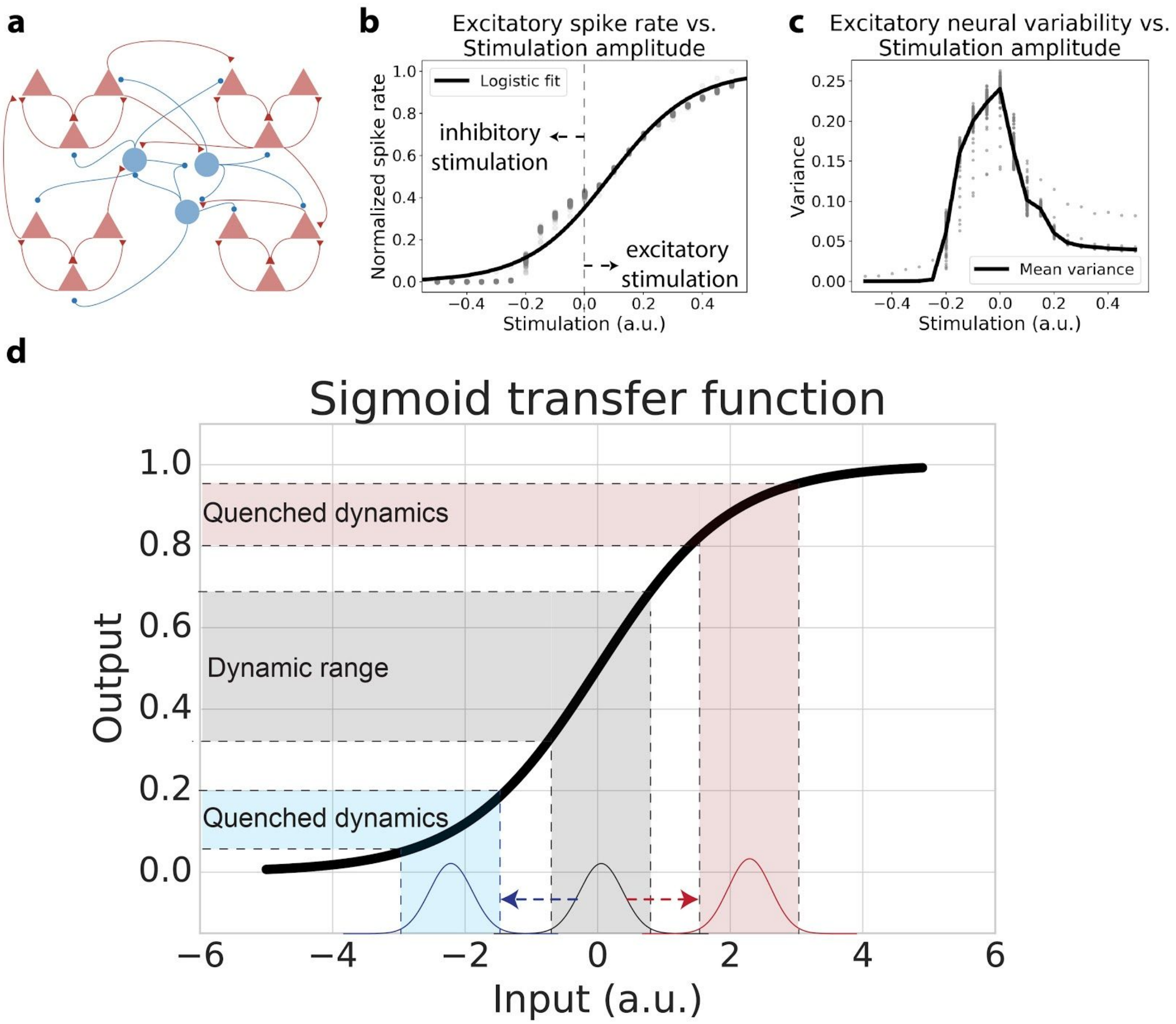

Fig 6. Inferring the mean-field transfer function of a neural population with a balanced spiking model with clustered excitatory connectivity. a) Schematic illustration of the balanced spiking model with clustered excitatory connections. Network architecture and parameters are identical to those reported in (Litwin-Kumar and Doiron 2012). Red triangles indicate excitatory cells, blue circles indicate inhibitory cells. **b)** The population spike rate (excitatory cells only) subject to inhibitory regulation. We systematically stimulated a subset of the neural population and measured the corresponding mean excitatory spike rate. Spike rates were normalized between 0 and 1. Excitatory stimulation was implemented by stimulating 400 excitatory neurons, and inhibitory stimulation was implemented by stimulating 400 inhibitory neurons. Spiking statistics were calculated across 30 trials, with each point in the scatter plot indicating a different 50ms time bin. **c)** Population neural variability (excitatory cells only), as a function of input stimulation. **d)** Based on panel b, we approximated the mean field neural transfer function as a sigmoid. A sigmoid transfer function produces optimal input-output dynamics for a narrow range of inputs (gray). The same input distribution mean shifted by some excitatory/inhibitory stimulation produces a quenched dynamic range.

In addition, in the revised manuscript we have added a large-scale network model comprising of 300 mean-field units (S11 Fig). We included 4 large-scale network models: 1) 300 unit network with random connections (excitatory efficacies); 2) 300 unit network with random connections (80% excitatory, 20% inhibitory efficacies); 3) 300 unit network with clustered connections (100% excitatory efficacies); 4) 300 unit network with clustered connections (80% excitatory, 20% inhibitory efficacies). Models #2 and #4 account for the possibility of an excitatory drive impinging primarily on local inhibitory neurons. In all models, we found that correlations primarily decreased in the presence of stimulation.

For additional details on on the model, see response #19 below.

****

- Page 16 first para: "We found a highly negative association between mean and variance under experimental perturbation, suggesting that at the mean-field level, mean and variance cannot be mechanistically dissociated" -- This is correct when we assume that the operating point of the network is right at the middle of the Sigmoid function. Authors have conveniently chosen a range of 0-1Hz for activity. In reality a network is operating at 1-2Hz (per neuron) baseline and stimulus evoked activity can push the network to very high firing rates transiently but certainly up to 20Hz in steady state. But cortical network activity will not reach saturation range -- if it does please provide suitable references. So the input induced reduction in variability while correct in the chosen model, does not make sense for a cortical network -- biological neural networks, irrespective of the spatial scale we observe them do not work in a saturation regime during evoked activity whereas in this model evoked activity induced reduction in variability is reached when the network moves towards saturation. Authors have also ignored their own data which shows that multi-unit firing rates different by a small amount in evoked and ongoing activity states -- that is even with the chosen Signoid function input induced shift in the operating point by input will be only 0.11 Hz.

****

18. We apologize to the reviewer for not being clearer. The specific operating range the sigmoid transfer function was not intended to be 0-1Hz, but rather the normalized (min and max) of the neural population’s spike rate. (In other words, 0 and 1 represent the minimum and maximum firing rate for a given neural population.) This was chosen because the typical mean-field spike rates for neural populations likely varies with location/cortical area, and so accounting for regional idiosyncrasies would not provide a generalizable model. While this was explicitly labeled for the Fig. 6b, the axes were not explicitly labeled for Fig. 6d, Fig. 7a, Fig. 8a,c. We have revised those figures to ensure that ‘normalized spike rate’ was explicitly placed in the y-axis labels.

Relatedly, R3 raises an interesting point, suggesting that biologically, neurons do not typically reach a saturating spiking regime. In general, we agree that many neurons (particularly in our data set) do not reach a saturating spiking regime. Moreover, our intention behind the sigmoid transfer function (particularly the upper bound of the sigmoid) was not to model saturating

spiking activity, but rather excitatory spiking activity *subject to inhibitory regulation*. In other words, the upper bound of the sigmoid transfer function implicitly models inhibitory stabilization to prevent runaway excitation (i.e., epilepsy) or saturation. We apologize that this was not made clearer. The general intuition was taken from the concept of inhibitory stabilization, providing an 'effective' upper bound on stimulus-driven activity (Hennequin, Agnes, and Vogels 2017; Ahmadian and Miller 2019).

The use of the balanced spiking model in Fig 6A justified the use of a sigmoid transfer function, since we measured the mean-field excitatory activity subject to feedback inhibition under a continuous range of stimulation (Fig 6B). Thus, though biologically neurons don't necessarily reach saturating levels of activity, the sigmoid function (at the population level) can still provide an accurate I/O description of bounded excitatory output given some stimulus-drive.

We have revised the text in the Results section introducing the sigmoid transfer function, as well as parts of the Discussion section to ensure further clarity.

Results (p. 14):

"We note that the upper bound on the sigmoid transfer function (Fig 6D) is likely due to inhibitory feedback on excitatory activity rather than the true saturating spiking regime in neurons. This is because excitatory neurons in a local population typically do not reach a saturating spiking regime even for strong visual stimuli (Priebe and Ferster 2008), and instead reach an upper bound due to strong inhibitory stabilization preventing runaway excitation (Hennequin et al. 2018)."

Discussion (p. 22):
"We propose that a sigmoid transfer function is an effective model of the activation dynamics of large cortical areas. This was based on causally manipulating a locally balanced E-I circuit with clustered excitatory connectivity (Litwin-Kumar and Doiron 2012). The sigmoid transfer function provides a simplification of mean-field neural dynamics that captures excitatory dynamics subject to inhibitory regulation, where feedback inhibition is implicitly modeled by the saturation of the sigmoid function. (We note that the saturation of the sigmoid function does not represent the saturating spiking regime, since spiking saturation does not typically occur *in vivo (Priebe and Ferster 2008)*)."

****

- The bidirectionally coupled network of two neuron mass models is way too simple to comment on the FC measured across many brain regions. For a start they have ignored the fact that there is a sizeable delay in inter-brain region connectivity. This model is a gross oversimplification. And even with this simplification, the mechanism of stimulus induced reduction in correlation is not becoming apparent. There is no definition of correlation or variance and neither these are related to the characteristic time scales. Even if we accept the arguments authors have made, it

is important to show that this argument holds true when we consider a network of N (=no of voxels in fMRI or brain regions) neuron mass models, not just 2.

****

19. The neural mass model was intended to be the simplest possible model to illustrate the phenomenon of interest, facilitating explanation and interpretation of the phenomenon. This "simplest possible model" approach is a common strategy used in computational modeling to clarify complex phenomena. This approach was complementary to 1) the complexity of the spiking neural model (i.e., less simplification of the underlying biology), and 2) the empirical results (i.e., no simplification of the underlying biology).

Though larger models may provide additional insight (e.g., insight into how network organization may affect correlations), these models contain a huge parameter space, potentially obfuscating the critical dynamical mechanisms that contribute to correlation changes. Using our simple model, we were able to pinpoint a dynamical mechanism - the characteristic time scale near a fixed point attractor - that explains changes to simulated variability and correlations independent of complex network parameters. Importantly, while we pinpoint a specific dynamical mechanism that governs these statistical changes, we also acknowledge a sigmoid transfer function (at the mean-field) can be implemented with a range of biological (and network) mechanisms, such as clustered excitatory connectivity (Litwin-Kumar and Doiron 2012), and/or a transition from loose to tight E-I balance (Hennequin et al. 2018). We note that the mechanisms we suggest are theoretically consistent with many of the explanations in other papers, where variability is reduced due to strengthening dynamics around a stable fixed point. We characterized this in the Discussion section (p. 22):

"The quenching of output variability can be explained by different biological mechanisms, such as clustered excitatory connectivity in local circuits, tightening of E-I balance due to inhibitory feedback, neural adaptation, and/or irregular synaptic vesicle release (Doiron et al. 2016; Rosenbaum, Rubin, and Doiron 2012; Hennequin et al. 2018; Litwin-Kumar and Doiron 2012; Deco and Hugues 2012). The manifestation of these biological mechanisms can be summarized at the mean-field by the reduction of the response variability due to the decreased slope in the sigmoid transfer function during highly active or inactive states."

However, to ameliorate any concerns that the mechanisms observed in a simple case would not scale appropriately to a large-scale network, we have provided additional numerical simulations with n=300 units. Given that the space of possible models increases in large-scale models (and an exhaustive overview of all possible scenarios would be beyond the scope of this study), we limited our analysis to include simulations with only sparse and random connectivity matrices with different connectivity weights. Below (and in the manuscript), we show the results demonstrating that at the large-scale, stimulus-driven activity reduces global variability and correlations:

On variability changes in large-scale networks (Results, p.16):

“We also generalized the findings from our minimal (single region) model to large-scale firing rate models (with 300 regions), where we found variability decreases during task-evoked states in both network models with random structural connections and clustered structural connections (SFig 11). We demonstrated this for network models with excitatory connections only, as well as networks with both E and I connections.”

On correlation changes in large-scale networks (Results, p. 20):
“To ensure that our findings in simplified two node networks would scale to large-scale network models, we simulated large-scale firing rate models (with 300 regions). We found correlation decreases during task-evoked states in both network models with random structural connections and clustered structural connections (SFig 11), suggesting that the mechanisms we identified in these minimal models likely scale to the larger networks. We demonstrated this for network models with excitatory connections only, as well as networks with both E and I connections.”

S11 Fig, p. 53:

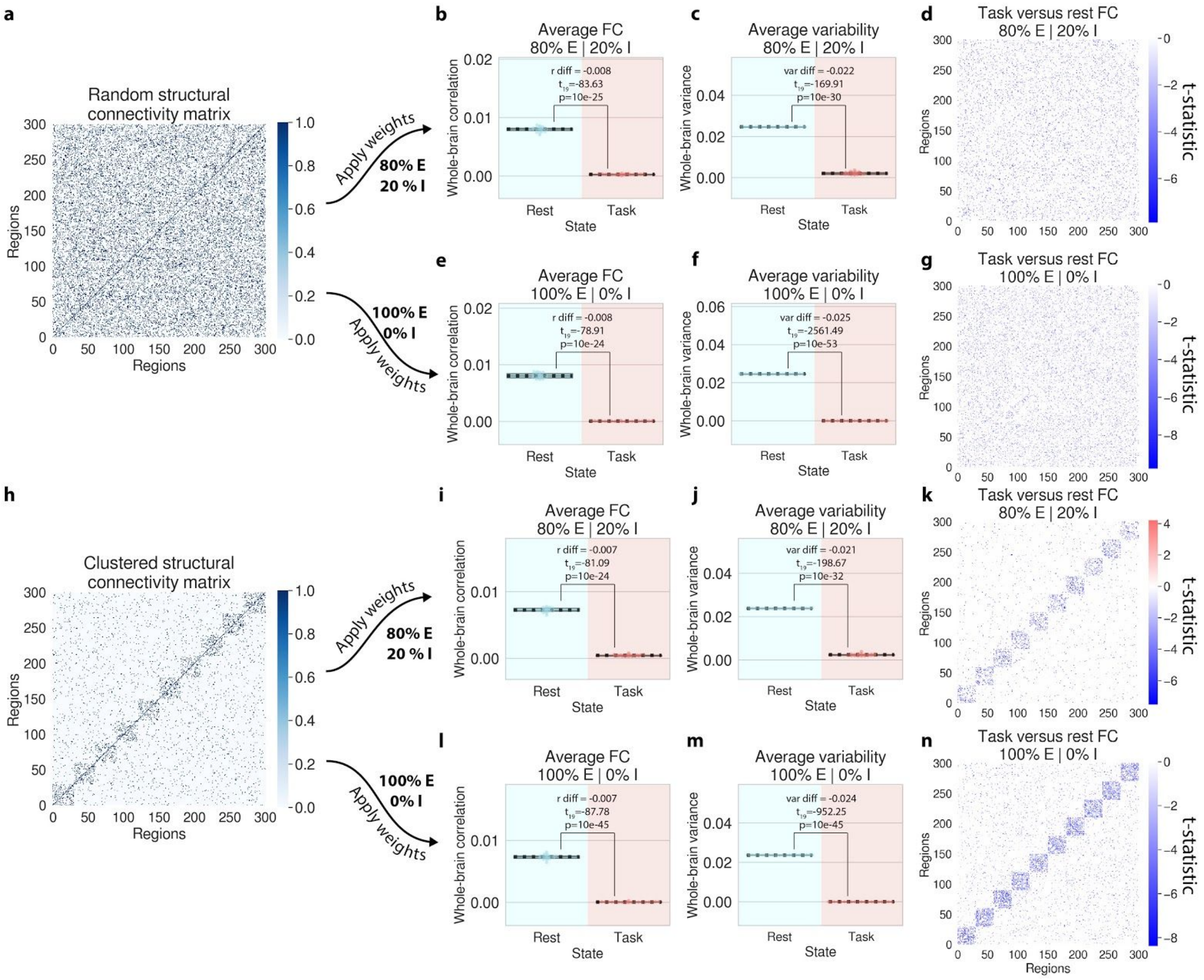

“S11 Figure. Variability and correlations are quenched in large-scale network models (300 regions) with both random and clustered structural connections. For each structural connectivity

matrix, we randomly sampled synaptic weights from a normal distribution with either 100% E connections (given evidence that most long-range connections are excitatory, $\mu = 1.0,\ \sigma = 0.2$ Joglekar et al., 2018), or 80% E and 20% I connections ($\mu = 1.0,\ \sigma = 1.2$). For each network model (4 in total), we simulated 20 subjects for 10 seconds each (100ms sampling rate). For simplicity, during the task state, all units were stimulated with a fixed input. **a)** Random structural connectivity matrix (20% connectivity density) for an example subject. **b)** The average across all pairwise correlations during the rest and task states for the network model with 80% E and 20% I connections. The rest state exhibits higher correlations than the task state. **c)** The variability (variance across time) averaged across brain regions during the rest and task states for the network model with 80% E and 20% I connections. The rest state exhibits higher variability than the task state. **d)** The task minus rest FC matrix (correlation difference) between all 300 regions. Correlations decreased from rest to task states. **e-g)** The same analyses as **b-d**, but using only excitatory connections only. **h)** Clustered structural connectivity matrix (10 communities, 20% within-community density, 3% out-of-community density). **i-k)** The same analyses as **b-d**, but using the clustered connectivity matrix with 80% E and 20% I connections. **l-n)** The same analyses as **b-d**, but using the clustered connectivity matrix with 100% E connections. Boxplots indicate the interquartile range of the distribution, dotted black line indicates the mean, and the distribution is visualized using a swarm plot. Plots **d, g, k, n,** were corrected for multiple comparisons and thresholded using an FDR-corrected p<0.05."

Methods of the 300-unit network model (Methods, p. 35-36):

"Model: 300 unit firing rate model

To verify the findings observed in our minimal models would scale to larger networks (one and two-dimensional models), we included a 300 region mean-field firing rate model. We chose 300 regions given that most whole-brain human atlases contain 200-400 cortically defined parcels (Glasser et al. 2016; Power et al. 2011; Schaefer et al. 2018). Our model followed the same equations as in our minimal model, though inter-area weights were appropriately scaled relative regional self-coupling parameters. Specifically, the network dynamics obeyed the equations

$$\tau_i \frac{dx_i}{dt} = -x_1 + f(w_{ii}x_i + \sum_{j\neq i}^{300} w_{ji}x_j + b_i + s_i + I_i) \qquad (10)$$

where x_i describes the activity of each population, and all other variables are as described above. Inter-regional coupling was set to be greater than local coupling (2:1 ratio), given evidence from previous studies that global coupling is greater than local coupling (Deco et al. 2013; Ito et al. 2017; M. W. Cole, Ito, et al. 2016). Specific parameters for this network model were specified such that: $w_{ii} = 1$, the mean of inter-region coupling parameters was $\sum_{j\neq i}^{300} w_{ji} = 2$, $b_i = -2$, $\tau_i = 0.1$. I_i was sampled from a Gaussian distribution with mean 0 and standard deviation 1. $s_i = 0$ during rest state and $s_i = 1$ during task state.

In total, we ran simulations for two classes of network models: a network with random connections and a network with clustered communities (S11 Fig). For the random network, we randomly sampled connections with 20% probability rate between all pairs of regions. For the clustered network model, we generated 10 communities of 30 nodes each. Regions within the

community had a 20% probability rate for establishing a connection. Between-community connections had a 3% probability rate for establishing a connection.

For each class of network (random or clustered), we weighted connections with either positive weights only (i.e., only E connections) or both positive and negative weights (i.e., both E and I connections. For the E-only network, weights were sampled from a normal distribution with parameters $\mu = 1,\ \sigma = 0.2$. For the network with both E and I weights (80% E, 20% I), weights were sampled from a normal distribution with parameters $\mu = 1,\ \sigma = 1.2$.

Both the rest and task state simulation was run for 10 seconds each and sampled at 100ms. For each group analysis (S11 Fig), we simulated 20 subjects worth of data. The full model code will be made publicly available, and was written in python 3.7.3."

R3 also makes a good point regarding the lack of inter-area synaptic delay in our model. As mentioned above, we opted to exclude synaptic delay parameters in our neural mass models due to the increased complexity (adding additional dynamic variables), which would make detailed dynamical systems analyses difficult. Instead, we showed in our balanced spiking model (which contains synaptic delays) that variability reductions occur during stimulus-driven activity even with a synaptic delay (Fig. 6b,c). Moreover, the time scale of synaptic dynamics is significantly faster (i.e., <10ms) than the time scale of variability/correlation changes we analyzed (>500ms in our spiking data and fMRI data). Because inter-areal synaptic delays operate on a much faster timescale than the variability/correlation reductions we were interested in, we believe that our model simplification (i.e., exclusion of synaptic delays) was appropriate.

Other studies have characterized inter-area neuron-to-neuron correlation changes using detailed biophysical models (Huang et al. 2019). We have referenced this paper (which offers a biophysical interpretation of correlation changes) in the Discussion section (p. 22). We wanted to offer a different perspective to contrast those models, using simple models and providing dynamical (rather than biophysical) explanations.

"Though other detailed spiking models have offered biophysical mechanisms for inter-area spiking correlations (Huang et al. 2019), we focused here on simplified dynamical systems explanations for correlation and variability changes at the mean-field. It will be important for future work to directly investigate how lower-level biophysical mechanisms map onto model descriptions at the mean-field." (p. 22, Discussion)

****

Minor:

Methods: I am confused about the spike rates. In the first para of the methods subsection Spiking data: Task vs rest variability analysis explains how mean responses were subtracted from each trial. But then in the very next para they have used expression such as 'mean firing

rate'. Where is the change in the firing rate used and displayed? And I hope that in eq 1 it is the true rate not a mean subtracted rate.

****

21. We apologize for any confusion the text has caused. We used ‘average’/’mean’ to refer to both the fact that the spikes within a cortical area were averaged *across neurons* (i.e., mean-field) in addition to the cross-trial mean. To simplify this text, we removed all instances of ‘average’/’mean’ except to refer to the fact that when computing the cross-trial variance, the cross-trial mean (of firing rate) was also removed for each condition separately. Text below (p. 24):

“We computed the variance across 25 consecutive trials using the spike rate from each cortical recording during either the ITI or the task cue period. This was repeated for all trials for each subject. We used across-trial variance to calculate variability rather than Fano factor (Churchland et al. 2010). This choice was due to the insight from our model illustrating that the mean-evoked activity and the corresponding variance interact in a nonlinear manner, and that the Fano factor is computed as the variance over the mean. Cross-trial variance was computed as

$$Var = \sum_{trial=x}^{x+n} \frac{(r_{trial} - \bar{r})^2}{n-1} \quad (1)$$

Where $n = 25$ trials, r_{trial} reflected the spike rate of each trial, and $\bar{r}$ is the cross-trial mean firing rate for the task condition (i.e., either the cross-trial mean firing rate during the color or motion task cue period).”

We clarify that $\bar{r}$ reflects the cross-trial firing rate for a given task condition (e.g., motion or color task).

****

- Authors wrote in the introduction "In the animal spiking literature," This is a rather sloppy expression and language. Humans are animals as well.

****

22. We have revised this sentence accordingly (p. 3):

“In the non-human primate (NHP) spiking literature, neural correlations have been measured by computing the correlation between the spike rate of two or more neurons and is commonly referred to as the spike count correlation (or noise correlation) [11].”

****

- Figure 2: Could authors display the results as a scatter diagram with x-axis as the firing rate in resting state and y-axis as the firing rate in task related state. This way we can see how firing rate at individual channels changed in task related state. In current display scheme it is not each

to see if activity at individual channel showed significant decrease or not. Same scatter diagram can be rendered for panels c and d.

****

23. As requested, we have included a scatter diagram of data in S15 Fig (below). However, we reiterate that the data represent mean-field approximations of the spiking dynamics (averaging the spiking activity from each recording site), and that each point reflects a set of 25 contiguous trials (ITI and task cue period, where ITI precedes the task cue period), as reported in the Methods section. We have referenced this Supplementary Figure in the caption of Fig 2.

Fig 2 caption (p. 6-7): "Scatter plot visualizations of **b-d** can be found in S15 Fig."

S15 Figure (p. 58):

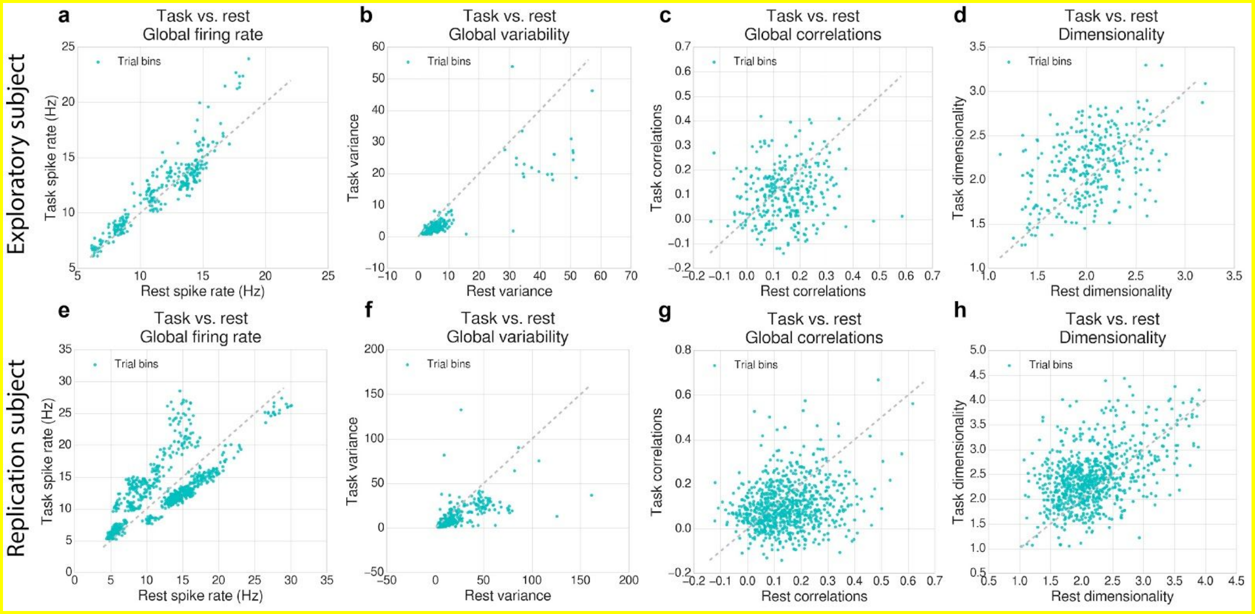

S15 Fig. Scatter plot representations of averaged neural statistics (firing rate, variability, correlations, dimensionality) during rest and task state periods. All data are identical to those reported in Fig 2b-d, Fig 5b, and S1b-d Fig, but are visualized as a scatter plot. In each scatter plot, every point reflects the statistic (mean, variance, correlations, dimensionality) estimated across a bin of 25 contiguous trials. (Rest periods were defined as the ITI preceding the task cue onset.) Statistics were averaged across all recording sites, and included all recording sessions. **a)** The firing rate (averaged across six cortical areas) for task (y-axis) and rest (x-axis) states. **b)** The variance (averaged across six cortical areas) for task (y-axis) and rest (x-axis) states. **c)** Correlations (averaged across all pairwise correlations) for task (y-axis) and rest (x-axis) states. **d)** Dimensionality (i.e., participation ratio) of all six cortical areas during task (y-axis) and rest (x-axis) states. **e-h)** The same as **a-d**, but for the replication subject.

****

- Figure 2: The caption says that panel b shows average spike rate across all recordings but the figure actually shows Spike rate change.

****

24. We have updated Fig 2B to show the true firing rate (without removal of the mean) for the rest (ITI) and task (cue) periods. We have also updated the corresponding figure for the replication subject (S1 Fig).

****

- Figure 2: In panels b-d what does each single dot mean? Is it average of a single session?

****

25. We have updated the caption to specify that each dot represents the statistic (e.g., firing rate) across 25 consecutive trials (which is also described in the Methods) (p. 6):

"**b)** We calculated the average spike rate across all recordings during the rest period (ITI) and task period (task cue), across trials. Each data point reflects the firing rate across 25 consecutive trials. **c)** We calculated the cross-trial spiking variance for each region during task and rest states, and then averaged across all regions. Each data point reflects the spiking variance across 25 consecutive trials. **d)** We calculated the average cross-trial neural correlation for task and rest states between all pairs of recorded brain regions. (Spike rates were averaged within each cortical area.) Each data point reflects the correlation across 25 consecutive trials."

****

- Page 12: Authors write "We hypothesized that decreases in neural variability and correlations are related to task-evoked activity." But this is not really a hypothesis. Authors must explain why they hypothesize so and what kind of relationship they expect? Same applies to several other hypotheses mentioned in the manuscript. For instance, there is a hypothesis mentioned in the last para on page 12 but that refers to information representation. But on page 13 first para authors start a sentence as 'Consistent with our hypothesis....' But this refers to dimensionality not to information representation. Dimensionality and information representation are two different concepts even if they stem from the fact that correlations are reduced. Better information representation does not predict high-dimensionality.

****

26. We have removed the reference to the hypothesis, and instead rephrased the text to indicate that the analysis was motivated from findings in the preceding Results sections. We have also referenced previous work that has shown that during a single task, task-evoked activity was negatively associated with variability (He 2013). We replicated these findings in the

additional task states included in this study, as well as extend this relationship to activity and correlation reductions (p. 12):

"We showed that task states widely reduced neural variability and correlations. We sought to extend this work to directly demonstrate that decreases in neural variability and correlations are associated with changes in task-evoked activity levels. To provide evidence for this hypothesis, we computed the mean task-evoked activity (averaging across all regions). We found that the global activity was significantly greater than baseline across different task states (exploratory cohort, $t_{175} = 6.46$, p<10e-9; replication cohort, $t_{175} = 12.63$, p<10e-25), demonstrating that decreases in neural variability and FC were accompanied by global increases in task-evoked activity.

Previous work has shown that regions that have strong task activations (i.e., the magnitude of the task-induced activation, positive or negative) tend to have greater variability reductions (He 2013). (Task activation magnitudes reflect the deflection of the BOLD activity relative to baseline, or the inter-block interval.) We sought to replicate this effect in the current data set, while extending those results to demonstrate that more task-active regions also tend to reduce their FC during task states."

We have also re-phrased our wording regarding the relationship between neural dimensionality and cognitive information. Moreover, we have directly linked our empirical hypothesis (i.e., that task state activity increases dimensionality) to predictions made by two previous computational modeling studies (Abbott, Rajan, and Sompolinsky 2011; Deco et al. 2014). We have made clear that an increase in dimensionality does not necessitate a change to cognitive information (p. 13):

"Here we sought to better characterize the information-theoretic implication of a global reduction in neural correlations. In particular, consistent with previous large-scale computational models that have predicted increased dimensionality with stimulus-driven activity (Abbott, Rajan, and Sompolinsky 2011; Deco et al. 2014), we hypothesized that reductions in neural correlations increase the effective dimensionality across units by suppressing background spontaneous activity/noise. While this increased dimensionality may potentially supports more robust information representations, we acknowledge that a change in neural dimensionality does not necessitate an improvement (or change) in cognitive information representation (Averbeck, Latham, and Pouget 2006), and that future studies will need to evaluate the relationship between neural dimensionality and cognitive content."

****

Also a short remark on the correlation and information representation -- authors have cited the Averbeck review for this. But please note that the correlation mentioned in the context of information representation were measured at 10s of milisec and FC of FMRI is measured at

much slower time scales than that. Information representation across brain areas is not the same as across neurons in a single local network.

****

We agree that information representations in a local network likely differ to information representations across large-scale neural systems. However, as mentioned above in responses #8 and #9, we have tried to match spatial and temporal resolutions in both our NHP and fMRI data as best we could. And while the Averbeck review focused on information representations at much finer spatial scales, we believe our focus on across large mean-field areas provides a unique perspective that has previously been difficult due to limitations in simultaneously collecting spiking data from multiple cortical areas.

****

- Figure 7c,d: The two variables do not seem to be 'perfectly correlated'

****

The rank correlation of the two variables is r=0.9996 (rounded to 1.0). We have adjusted the language in the caption (p. 17):

"The characteristic time scale across all fixed points are nearly perfectly correlated with the neural variability of the simulated time series across all fixed inputs (rank correlation = 0.9996)."

We have also revised in the in-text reference to this Figure and analysis (p. 18):

"Again, we found a nonlinear relationship between the amplitude of the stimulus and the characteristic time scale of the neural population (Fig 7D), and found that the characteristic time scale explained nearly 100% of the variance (rho=0.9996) of the simulated stimulus-evoked variability (Fig 7C)."

****

- Authors write on Page 3 "...the mechanism underlying these reduced correlations remains unclear." This is not correct. The review by Doiron provides an exhaustive overview of various mechanisms that may underlie reduction in correlation and variance e.g. clustered connectivity (Litwin-Kumar and DOiron 2012, Deco and Higher 2012), tight and detailed balance of excitation and inhibition (Hennequin et al.) or stimulus/input (Bujan et al. 2015)

****

Indeed, while the review by Doiron et al. 2016 provides an exhaustive overview of various mechanisms that may underlie reductions in spike count correlations, most of these explanations are biophysical mechanisms at the level of local circuits. We focus here on mean-field, inter-area correlation mechanisms that are relevant to fMRI BOLD signals and mean-field spike rates.

While the Deco and Hughes 2012 paper provides mechanistic explanations for variability reductions that apply at the mean-field level, they use a local spiking neural network model. More importantly, the proposed mechanisms are limited to explaining neural variability and were not extended to mechanisms explaining inter-area correlations/functional connectivity. We specifically wanted to extend these findings to identify mechanisms that could explain *inter-area mean-field correlation changes*, which could facilitate translation to the level of functional connectivity in fMRI data. In fact, we were inspired by these papers such that we used the model from Litwin-Kumar and Doiron 2012 to justify the use of a sigmoid transfer function to describe mean-field population dynamics. The publications R3 has mentioned (i.e., Litwin-Kumar and Doiron 2012, Doiron et al. 2016, Deco and Hughes 2012, Hennequin et al. 2018) have been cited throughout the paper, but most prominently in the Discussion. We thank the reviewer for bringing our attention to Bujan et al. 2015. We have now included a reference to that study as well.

Discussion (p. 22):
"The quenching of output variability can be explained by different biological mechanisms, such as clustered excitatory connectivity in local circuits, tightening of E-I balance due to inhibitory feedback, the statistics of feedforward inputs, neural adaptation, and/or irregular synaptic vesicle release (Bujan, Aertsen, and Kumar 2015; Deco and Hugues 2012; Doiron et al. 2016; Hennequin et al. 2018; Litwin-Kumar and Doiron 2012). The manifestation of these biological mechanisms can be summarized at the mean-field by the reduction of the response variability due to the decreased slope in the sigmoid transfer function during highly active or inactive states."

References

Abbott, Larry F., Kanaka Rajan, and Haim Sompolinsky. 2011. "Interactions between Intrinsic and Stimulus-Evoked Activity in Recurrent Neural Networks." *The Dynamic Brain: An Exploration of Neuronal Variability and Its Functional Significance*, 1–16.

Ahmadian, Y., and K. D. Miller. 2019. "What Is the Dynamical Regime of Cerebral Cortex?" *arXiv Preprint arXiv:1908.10101*. http://arxiv.org/abs/1908.10101.

Averbeck, Bruno B., Peter E. Latham, and Alexandre Pouget. 2006. "Neural Correlations, Population Coding and Computation." *Nature Reviews. Neuroscience* 7 (May): 358.

Brincat, Scott L., Markus Siegel, Constantin von Nicolai, and Earl K. Miller. 2018. "Gradual Progression from Sensory to Task-Related Processing in Cerebral Cortex." *Proceedings of the National Academy of Sciences* 115 (30): E7202 LP – E7211.

Bujan, Alejandro F., Ad Aertsen, and Arvind Kumar. 2015. "Role of Input Correlations in Shaping the Variability and Noise Correlations of Evoked Activity in the Neocortex." *The Journal of Neuroscience: The Official Journal of the Society for Neuroscience* 35 (22): 8611–25.

Churchland, Mark M., Byron M. Yu, John P. Cunningham, Leo P. Sugrue, Marlene R. Cohen, Greg S. Corrado, William T. Newsome, et al. 2010. "Stimulus Onset Quenches Neural Variability: A Widespread Cortical Phenomenon." *Nature Neuroscience* 13 (February): 369.

Cole, Michael, Danielle Bassett, Jonathan Power, Todd Braver, and Steven Petersen. 2014. "Intrinsic and Task-Evoked Network Architectures of the Human Brain" 83 (1). https://doi.org/10.1016/j.neuron.2014.05.014.
Cole, Michael W., Takuya Ito, Danielle S. Bassett, and Douglas H. Schultz. 2016. "Activity Flow over Resting-State Networks Shapes Cognitive Task Activations." *Nature Neuroscience* 19 (12): 1718–26.
Cole, Michael W., Genevieve J. Yang, John D. Murray, Grega Repovš, and Alan Anticevic. 2016. "Functional Connectivity Change as Shared Signal Dynamics." *Journal of Neuroscience Methods* 259: 22–39.
Deco, Gustavo, and Etienne Hugues. 2012. "Neural Network Mechanisms Underlying Stimulus Driven Variability Reduction." *PLoS Computational Biology* 8 (3): e1002395.
Deco, Gustavo, Adrián Ponce-Alvarez, Patric Hagmann, Gian Luca Romani, Dante Mantini, and Maurizio Corbetta. 2014. "How Local Excitation-Inhibition Ratio Impacts the Whole Brain Dynamics." *The Journal of Neuroscience: The Official Journal of the Society for Neuroscience* 34 (23): 7886–98.
Deco, Gustavo, Adrián Ponce-Alvarez, Dante Mantini, Gian Luca Romani, Patric Hagmann, and Maurizio Corbetta. 2013. "Resting-State Functional Connectivity Emerges from Structurally and Dynamically Shaped Slow Linear Fluctuations." *The Journal of Neuroscience: The Official Journal of the Society for Neuroscience* 33 (27): 11239–52.
Doiron, Brent, Ashok Litwin-Kumar, Robert Rosenbaum, Gabriel K. Ocker, and Krešimir Josić. 2016. "The Mechanics of State-Dependent Neural Correlations." *Nature Neuroscience* 19 (February): 383.
Duff, Eugene P., Tamar Makin, Michiel Cottaar, Stephen M. Smith, and Mark W. Woolrich. 2018. "Disambiguating Brain Functional Connectivity." *NeuroImage* 173: 540–50.
Friston, K. J., L. Harrison, and W. Penny. 2003. "Dynamic Causal Modelling." *NeuroImage* 19 (4): 1273–1302.
Glasser, Matthew F., Timothy S. Coalson, Emma C. Robinson, Carl D. Hacker, John Harwell, Essa Yacoub, Kamil Ugurbil, et al. 2016. "A Multi-Modal Parcellation of Human Cerebral Cortex." *Nature* 536 (7615): 171–78.
Gonzalez-Castillo, Javier, and Peter A. Bandettini. 2017. "Task-Based Dynamic Functional Connectivity: Recent Findings and Open Questions." *NeuroImage*, no. May: 1–8.
Gordon, Evan M., Timothy O. Laumann, Adrian W. Gilmore, Steven E. Petersen, Steven M. Nelson, Nico U. F. Dosenbach, Evan M. Gordon, et al. 2017. "Precision Functional Mapping of Individual Human NeuroResource Precision Functional Mapping of Individual Human Brains." *Neuron*, 1–17.
He, Biyu J. 2013. "Spontaneous and Task-Evoked Brain Activity Negatively Interact." *The Journal of Neuroscience: The Official Journal of the Society for Neuroscience* 33 (11): 4672–82.
Hennequin, Guillaume, Everton J. Agnes, and Tim P. Vogels. 2017. "Inhibitory Plasticity: Balance, Control, and Codependence." *Annual Review of Neuroscience* 40 (July): 557–79.
Hennequin, Guillaume, Yashar Ahmadian, Daniel B. Rubin, Máté Lengyel, and Kenneth D. Miller. 2018. "The Dynamical Regime of Sensory Cortex: Stable Dynamics around a Single Stimulus-Tuned Attractor Account for Patterns of Noise Variability." *Neuron* 98 (4): 846–60.e5.
Huang, Chengcheng, Douglas A. Ruff, Ryan Pyle, Robert Rosenbaum, Marlene R. Cohen, and Brent Doiron. 2019. "Circuit Models of Low-Dimensional Shared Variability in Cortical Networks." *Neuron* 101 (2): 337–48.e4.
Huth, Alexander G., Shinji Nishimoto, An T. Vu, and Jack L. Gallant. 2012. "A Continuous

Semantic Space Describes the Representation of Thousands of Object and Action Categories across the Human Brain.” *Neuron* 76 (6): 1210–24.
Ito, Takuya, Kaustubh R. Kulkarni, Douglas H. Schultz, Ravi D. Mill, Richard H. Chen, Levi I. Solomyak, and Michael W. Cole. 2017. “Cognitive Task Information Is Transferred between Brain Regions via Resting-State Network Topology.” *Nature Communications* 8 (1): 1027.
Joglekar, Madhura R., Jorge F. Mejias, Guangyu Robert Yang, and Xiao-Jing Wang. 2018. “Inter-Areal Balanced Amplification Enhances Signal Propagation in a Large-Scale Circuit Model of the Primate Cortex.” *Neuron* 98 (1): 222–34.e8.
Krienen, F. M., B. T. T. Yeo, and R. L. Buckner. 2014. “Reconfigurable Task-Dependent Functional Coupling Modes Cluster around a Core Functional Architecture.” *Philosophical Transactions of the Royal Society of London. Series B, Biological Sciences* 369 (1653): 20130526–20130526.
Litwin-Kumar, Ashok, and Brent Doiron. 2012. “Slow Dynamics and High Variability in Balanced Cortical Networks with Clustered Connections.” *Nature Neuroscience* 15 (11): 1498–1505.
Logothetis, N. K., J. Pauls, M. Augath, T. Trinath, and A. Oeltermann. 2001. “Neurophysiological Investigation of the Basis of the fMRI Signal.” *Nature* 412 (6843): 150–57.
Ma, Ying, Mariel G. Kozberg, Sharon H. Kim, Mohammed A. Shaik, Dmitriy Timmerman, and Elizabeth M. C. Hillman. 2016. “Resting‐State Hemodynamics Are Spatiotemporally Coupled to Synchronized and Symmetric Neural Activity in Excitatory Neurons.” *Proceedings of the National Academy of Sciences*. https://doi.org/10.1073/pnas.1525369113.
Miller, Paul. 2018. *An Introductory Course in Computational Neuroscience*. MIT Press.
Nakai, Tomoya, and Shinji Nishimoto. 2020. “Quantitative Models Reveal the Organization of Diverse Cognitive Functions in the Brain.” *Nature Communications* 11 (1): 1142.
Power, Jonathan D., Alexander L. Cohen, Steven M. Nelson, Gagan S. Wig, Kelly Anne Barnes, Jessica a. Church, Alecia C. Vogel, et al. 2011. “Functional Network Organization of the Human Brain.” *Neuron* 72 (4): 665–78.
Priebe, Nicholas J., and David Ferster. 2008. “Inhibition, Spike Threshold, and Stimulus Selectivity in Primary Visual Cortex.” *Neuron* 57 (4): 482–97.
Rosenbaum, Robert, Jonathan E. Rubin, and Brent Doiron. 2012. “Short-Term Synaptic Depression and Stochastic Vesicle Dynamics Reduce and Shape Neuronal Correlations.” *Journal of Neurophysiology* 109 (2): 475–84.
Ruff, Douglas A., and Marlene R. Cohen. 2014. “Attention Can Either Increase or Decrease Spike Count Correlations in Visual Cortex.” *Nature Neuroscience* 17 (11): 1591–97.
Schaefer, Alexander, Ru Kong, Evan M. Gordon, Timothy O. Laumann, Xi-Nian Zuo, Avram J. Holmes, Simon B. Eickhoff, and B. T. Thomas Yeo. 2018. “Local-Global Parcellation of the Human Cerebral Cortex from Intrinsic Functional Connectivity MRI.” *Cerebral Cortex* 28 (9): 3095–3114.
Siegel, Markus, Timothy J. Buschman, and Earl K. Miller. 2015. “Cortical Information Flow during Flexible Sensorimotor Decisions.” *Science* 348 (6241): 1352–55.
Siegel, Markus, Tobias H. Donner, and Andreas K. Engel. 2012. “Spectral Fingerprints of Large-Scale Neuronal Interactions.” *Nature Reviews. Neuroscience*, January. https://doi.org/10.1038/nrn3137.
