# Supplementary material for: Task-evoked activity quenches neural correlations and variability across cortical areas
Source: PLoS Comput Biol. 2020 Aug 3;16(8):e1007983. doi: 10.1371/journal.pcbi.1007983 (PMC7425988; doi:10.1371/journal.pcbi.1007983)
Supplement: Attachment — Submitted filename: ResponseToReviewers_2ndRound.pdf [file pcbi.1007983.s017.pdf]

Response to Reviewer comments #2: "Task-evoked activity quenches neural correlations and variability across cortical areas"

We thank the editor and the reviewers for the opportunity to re-submit a revised version of our manuscript. (Reviewer comments in italics. Highlighted text indicates revised portions of the manuscript.)

****

Reviewer #3: The authors have done a great job in addressing most of the concerns I raised in the previous round of reviews. The manuscript is a very good shape and has some very interesting results. There are a few minor issues that still bother me.

****

We are glad to hear that R3 is generally positive about the revised manuscript. Below, we address the remaining concerns.

****

I strongly object to the following statement in the introduction.
"These differing perspectives of the role of neural correlations in (large-scale) human imaging and (local circuit) animal neurophysiology studies appear to be incompatible. "

As I said in my previous comments, there is no reason why they should be consistent.
Both spiking activity and fMRI are brain's activity -- they have to be related. But not in a liner manner. It is a way non-linear relationship. That is, even if first moments of the spiking activity distribution and fMRI signals are related there is no reason to believe that other moments (second in this case) will also be. So I think author should remove the sentence. I think it is perfectly fine to quantify the relationship between noise correlations and functional connectivity -- that's a good enough motivation.

****

We have removed this sentence, and replaced it with (Introduction, p. 3):
"Here we sought to quantify the relationship between neural correlations (i.e., FC) in large-scale human imaging and (local circuit) animal neurophysiology (spike count correlations)."

****

Similarly, while mechanistic basis of FC in fMRI remains unclear the same is not correct for spiking activity NC and reduction in NC. A number of papers have discussed this (e.g. Tetzlaff et al. 2012 PloS Comp., Renart et al. 2010 Science, Bujan et al. 2015 JNeurosci, Ecker et al. and several of papers from Dorion/Eric Shea-Brown groups).

****

Overall, we agree that the mechanistic basis for NC is more developed than FC. In fact, a primary motivation for this study was to draw insights and theory from the NC literature and apply them to FC in fMRI. (We also note that nearly all papers mentioned in the comment above have been already cited in the manuscript. We have now included Tetzlaff et al. (2012) in the manuscript as well.) We recognize some phrasing in the Introduction was poorly phrased (Introduction, p. 3): Original sentence: “Though the reduction in spike count correlations during task states has been linked to better information coding by the local neuronal population by suppressing shared spontaneous activity and reducing neural noise [10,22], the mechanism underlying these reduced correlations remains unclear.”

We have revised the paragraph that contained that sentence.
Introduction (p. 3):
“Studies in the local circuit literature using electrophysiological recordings in animals have characterized the correlation structure between neuron spikes across a range of task demands. These studies have found that the spike count correlation (or noise correlation) between neuron spikes generally decreases during task states, particularly for neurons that are responsive to the task (Cohen and Maunsell 2009; Ecker et al. 2010; Ruff and Cohen 2014; Pinto et al. 2019). Moreover, these empirical studies have been accompanied by theoretical work, which has suggested that the reduction in noise correlations may enhance information coding by suppressing shared spontaneous activity and reducing neural noise (Averbeck, Latham, and Pouget 2006; Cohen and Kohn 2011; da Silveira and Berry 2014; Aertsen et al. 1989; Doiron et al. 2016). Thus, the theoretical framework behind noise correlations may also provide a solid foundation from which to advance understanding of fMRI FC (Cohen and Kohn 2011; Doiron et al. 2016; Averbeck, Latham, and Pouget 2006; Aertsen et al. 1989).”

Passage citing Tetzlaff et al. (Discussion, p. 22): “The quenching of output variability can be explained by different biological mechanisms, such as clustered excitatory connectivity in local circuits, tightening of E-I balance due to inhibitory feedback, neural adaptation, and/or irregular synaptic vesicle release (Doiron et al. 2016; Rosenbaum et al. 2012; Hennequin et al. 2018; Litwin-Kumar and Doiron 2012; Deco and Hugues 2012; Tetzlaff et al. 2012).”

****

Next, I do not think that the difference in the temporal dynamics of the spiking activity signals and fMRI can be alleviated simply by down sampling the spiking activity signals. The difference in the dynamics also means that the mechanisms underlying the statistical properties also operate at different time scales. So comparing statistical properties of spiking activity and fMRI is a rather tricky task.

****

We recognize the difficulty in comparing spiking activity to fMRI activity, given the fundamental difference in the measured signals, timescales, and spatial resolutions. However, we maintain that the efforts put forth in the study (i.e., analyzing both fMRI and spiking activity together) are

an important first step to adopting an integrative perspective across these distinct neuroscience subfields. Additionally, much work has gone into demonstrating the relationship between spiking and fMRI BOLD activity, as we mentioned in our previous response (Logothetis et al. 2001; Ma et al. 2016).

Separately, in our computational model, we simulated fMRI BOLD dynamics by transforming the simulated spike activity through a nonlinear transformation that models neural hemodynamics (S6 Fig, S7 Fig) (Friston, Harrison, and Penny 2003). These model results, along with convergent empirical data that showed reductions in variability and correlations during task states in both data sets, seem to indicate the possibility of similar statistical properties of fMRI and spiking activity *in spite* of different timescales. However, we agree that our empirical data cannot rule out other potential mechanisms and explanations *in vivo*. Thus, we have qualified some of our interpretations in the Discussion, and have included another sentence in this revised manuscript to acknowledge these limitations.

Discussion (p. 21):
"Despite converging results, there are several key differences in our two empirical data sets. First, the time scale of fMRI BOLD activity is much slower than the NHP spiking activity. However, these differences were mitigated by measuring spiking variability across trials, which is comparable to the time scale of fMRI's sampling rate (in the hundreds of milliseconds). (We note, however, that downsampling spike data does not make it statistically equivalent to fMRI data.) In addition, our computational model results demonstrated that reductions in neural variability and correlations were preserved after nonlinearly transforming the spike rate signal to the fMRI BOLD signal with the Balloon-Windkessel model (Friston, Harrison, and Penny 2003), suggesting that the observed signal changes are likely due to the BOLD signal changes rather than MRI artifacts. Despite the computational model demonstrating that statistical properties of BOLD dynamics can be directly caused by spiking dynamics (via the hemodynamic transformation), it is difficult to rule out other possible *in vivo* explanations in the present study."

****

Finally, I am not convinced that the model is suitable to address the problem and even if this oversimplified model gives some insights, the question of scaling remains -- i.e. from two interacting populations to N interacting populations. It is a non-trivial task so I do not expect that the authors undertake this task for this manuscript. From my own experience, it almost never straightforward to translate insights from a two population network to N-population model.

We agree that it is often difficult to scale insights from a simple/minimal model to a larger model (e.g., 2-dimensions to n-dimensions). Moreover, the number of possible parameter choices become difficult to control for (e.g., community structure, regional heterogeneity, etc.). Though the question of scaling from a 2-dimensional to an n-dimensional model is an important and non-trivial question, we agree with R3 that a complete characterization of this issue is beyond the scope of the present study. Nonetheless, in the previous revision we scaled our

2-dimensional model to a 300-dimensional model. While not exhaustive, we have included some analyses and results in the Supplementary Information (S11 Fig). These preliminary results seem to indicate that insights from our minimal model scale well, and are also consistent with previously reported large-scale mean-field models (Deco and Hugues 2012; Ponce-alvarez et al. 2015).

However, we have qualified the Results statement where we mentioned that our simple model could scale to the n-dimensional case (Results, p. 16):

“We also generalized the findings from our minimal (single region) model to large-scale firing rate models (with 300 regions), where we found variability decreases during task-evoked states in both network models with random structural connections and clustered structural connections (SFig 11). We demonstrated this for network models with excitatory connections only, as well as networks with both excitatory and inhibitory connections. However, due to the large number of possible network models when scaling to n-dimensions, we constrained our analyses to only four network architectures, leaving a more complete analysis to future studies.”

Below, we have copied and pasted the original reply to the comment regarding the issue of scaling from 2 to n-dimensions.

Original review comment:

- The bidirectionally coupled network of two neuron mass models is way too simple to comment on the FC measured across many brain regions. For a start they have ignored the fact that there is a sizeable delay in inter-brain region connectivity. This model is a gross oversimplification. And even with this simplification, the mechanism of stimulus induced reduction in correlation is not becoming apparent. There is no definition of correlation or variance and neither these are related to the characteristic time scales. Even if we accept the arguments authors have made, it is important to show that this argument holds true when we consider a network of N (=no of voxels in fMRI or brain regions) neuron mass models, not just 2.

****

Original reply:

19. The neural mass model was intended to be the simplest possible model to illustrate the phenomenon of interest, facilitating explanation and interpretation of the phenomenon. This “simplest possible model” approach is a common strategy used in computational modeling to clarify complex phenomena. This approach was complementary to 1) the complexity of the spiking neural model (i.e., less simplification of the underlying biology), and 2) the empirical results (i.e., no simplification of the underlying biology).

Though larger models may provide additional insight (e.g., insight into how network organization may affect correlations), these models contain a huge parameter space, potentially obfuscating

the critical dynamical mechanisms that contribute to correlation changes. Using our simple model, we were able to pinpoint a dynamical mechanism - the characteristic time scale near a fixed point attractor - that explains changes to simulated variability and correlations independent of complex network parameters. Importantly, while we pinpoint a specific dynamical mechanism that governs these statistical changes, we also acknowledge a sigmoid transfer function (at the mean-field) can be implemented with a range of biological (and network) mechanisms, such as clustered excitatory connectivity (Litwin-Kumar and Doiron 2012), and/or a transition from loose to tight E-I balance (Hennequin et al. 2018). We note that the mechanisms we suggest are theoretically consistent with many of the explanations in other papers, where variability is reduced due to strengthening dynamics around a stable fixed point. We characterized this in the Discussion section (p. 22):

"The quenching of output variability can be explained by different biological mechanisms, such as clustered excitatory connectivity in local circuits, tightening of E-I balance due to inhibitory feedback, neural adaptation, and/or irregular synaptic vesicle release (Doiron et al. 2016; Rosenbaum, Rubin, and Doiron 2012; Hennequin et al. 2018; Litwin-Kumar and Doiron 2012; Deco and Hugues 2012). The manifestation of these biological mechanisms can be summarized at the mean-field by the reduction of the response variability due to the decreased slope in the sigmoid transfer function during highly active or inactive states."

However, to ameliorate any concerns that the mechanisms observed in a simple case would not scale appropriately to a large-scale network, we have provided additional numerical simulations with n=300 units. Given that the space of possible models increases in large-scale models (and an exhaustive overview of all possible scenarios would be beyond the scope of this study), we limited our analysis to include simulations with only sparse and random connectivity matrices with different connectivity weights. Below (and in the manuscript), we show the results demonstrating that at the large-scale, stimulus-driven activity reduces global variability and correlations:

On variability changes in large-scale networks (Results, p.16):

"We also generalized the findings from our minimal (single region) model to large-scale firing rate models (with 300 regions), where we found variability decreases during task-evoked states in both network models with random structural connections and clustered structural connections (SFig 11). We demonstrated this for network models with excitatory connections only, as well as networks with both E and I connections."

On correlation changes in large-scale networks (Results, p. 20):
"To ensure that our findings in simplified two node networks would scale to large-scale network models, we simulated large-scale firing rate models (with 300 regions). We found correlation decreases during task-evoked states in both network models with random structural connections and clustered structural connections (SFig 11), suggesting that the mechanisms we identified in these minimal models likely scale to the larger networks. We demonstrated this for

network models with excitatory connections only, as well as networks with both E and I connections."

S11 Fig, p. 53:

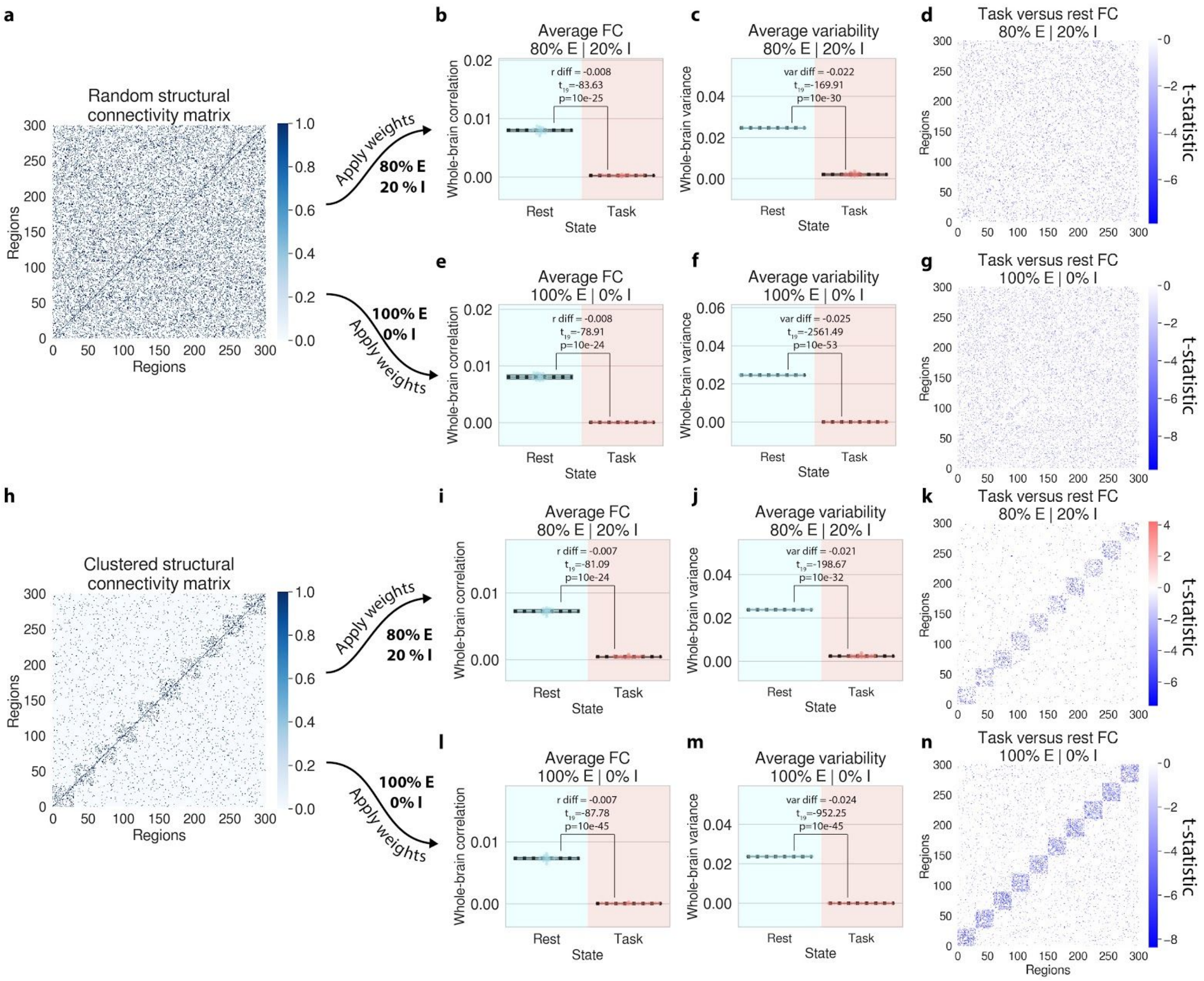

"S11 Figure. Variability and correlations are quenched in large-scale network models (300 regions) with both random and clustered structural connections. For each structural connectivity matrix, we randomly sampled synaptic weights from a normal distribution with either 100% E connections (given evidence that most long-range connections are excitatory, $\mu = 1.0,\ \sigma = 0.2$ Joglekar et al., 2018), or 80% E and 20% I connections ($\mu = 1.0,\ \sigma = 1.2$). For each network model (4 in total), we simulated 20 subjects for 10 seconds each (100ms sampling rate). For simplicity, during the task state, all units were stimulated with a fixed input. **a)** Random structural connectivity matrix (20% connectivity density) for an example subject. **b)** The average across all pairwise correlations during the rest and task states for the network model with 80% E and 20% I connections. The rest state exhibits higher correlations than the task state. **c)** The variability (variance across time) averaged across brain regions during the rest and task states for the network model with 80% E and 20% I connections. The rest state exhibits higher variability than the task state. **d)** The task minus rest FC matrix (correlation difference) between all 300 regions. Correlations decreased from rest to task states. **e-g)** The same analyses as **b-d**, but using only excitatory connections only. **h)** Clustered structural connectivity matrix (10 communities, 20% within-community density, 3% out-of-community density). **i-k)** The same analyses as **b-d**, but using the clustered

connectivity matrix with 80% E and 20% I connections. **l-n)** The same analyses as **b-d**, but using the clustered connectivity matrix with 100% E connections. Boxplots indicate the interquartile range of the distribution, dotted black line indicates the mean, and the distribution is visualized using a swarm plot. Plots **d, g, k, n,** were corrected for multiple comparisons and thresholded using an FDR-corrected p<0.05."

Methods of the 300-unit network model (Methods, p. 35-36):

"Model: 300 unit firing rate model

To verify the findings observed in our minimal models would scale to larger networks (one and two-dimensional models), we included a 300 region mean-field firing rate model. We chose 300 regions given that most whole-brain human atlases contain 200-400 cortically defined parcels (Glasser et al. 2016; Power et al. 2011; Schaefer et al. 2018). Our model followed the same equations as in our minimal model, though inter-area weights were appropriately scaled relative regional self-coupling parameters. Specifically, the network dynamics obeyed the equations

$$\tau_i \frac{dx_i}{dt} = -x_1 + f(w_{ii}x_i + \sum_{j \neq i}^{300} w_{ji}x_j + b_i + s_i + I_i) \quad (10)$$

where x_i describes the activity of each population, and all other variables are as described above. Inter-regional coupling was set to be greater than local coupling (2:1 ratio), given evidence from previous studies that global coupling is greater than local coupling (Deco et al. 2013; Ito et al. 2017; Cole et al. 2016). Specific parameters for this network model were specified such that: $w_{ii} = 1$, the mean of inter-region coupling parameters was $\sum_{j \neq i}^{300} w_{ji} = 2$, $b_i = -2$, $\tau_i = 0.1$. I_i was sampled from a Gaussian distribution with mean 0 and standard deviation 1. $s_i = 0$ during rest state and $s_i = 1$ during task state.

In total, we ran simulations for two classes of network models: a network with random connections and a network with clustered communities (S11 Fig). For the random network, we randomly sampled connections with 20% probability rate between all pairs of regions. For the clustered network model, we generated 10 communities of 30 nodes each. Regions within the community had a 20% probability rate for establishing a connection. Between-community connections had a 3% probability rate for establishing a connection.

For each class of network (random or clustered), we weighted connections with either positive weights only (i.e., only E connections) or both positive and negative weights (i.e., both E and I connections. For the E-only network, weights were sampled from a normal distribution with parameters $\mu = 1,\ \sigma = 0.2$. For the network with both E and I weights (80% E, 20% I), weights were sampled from a normal distribution with parameters $\mu = 1,\ \sigma = 1.2$.

Both the rest and task state simulation was run for 10 seconds each and sampled at 100ms. For each group analysis (S11 Fig), we simulated 20 subjects worth of data. The full model code will be made publicly available, and was written in python 3.7.3."

References

Aertsen, A. M., G. L. Gerstein, M. K. Habib, and Gwtcopgk Palm. 1989. “Dynamics of Neuronal Firing Correlation: Modulation of‘ Effective Connectivity.’” *Journal of Neurophysiology* 61 (5): 900–917.

Averbeck, Bruno B., Peter E. Latham, and Alexandre Pouget. 2006. “Neural Correlations, Population Coding and Computation.” *Nature Reviews. Neuroscience* 7 (May): 358.

Cohen, Marlene R., and Adam Kohn. 2011. “Measuring and Interpreting Neuronal Correlations.” *Nature Neuroscience* 14 (June): 811.

Cohen, Marlene R., and John H. R. Maunsell. 2009. “Attention Improves Performance Primarily by Reducing Interneuronal Correlations.” *Nature Neuroscience* 12 (12): 1594–1600.

Cole, Michael W., Takuya Ito, Danielle S. Bassett, and Douglas H. Schultz. 2016. “Activity Flow over Resting-State Networks Shapes Cognitive Task Activations.” *Nature Neuroscience* 19 (12): 1718–26.

Deco, Gustavo, and Etienne Hugues. 2012. “Neural Network Mechanisms Underlying Stimulus Driven Variability Reduction.” *PLoS Computational Biology* 8 (3): e1002395.

Deco, Gustavo, Adrián Ponce-Alvarez, Dante Mantini, Gian Luca Romani, Patric Hagmann, and Maurizio Corbetta. 2013. “Resting-State Functional Connectivity Emerges from Structurally and Dynamically Shaped Slow Linear Fluctuations.” *The Journal of Neuroscience: The Official Journal of the Society for Neuroscience* 33 (27): 11239–52.

Doiron, Brent, Ashok Litwin-Kumar, Robert Rosenbaum, Gabriel K. Ocker, and Krešimir Josić. 2016. “The Mechanics of State-Dependent Neural Correlations.” *Nature Neuroscience* 19 (February): 383.

Ecker, Alexander S., Philipp Berens, Georgios A. Keliris, Matthias Bethge, Nikos K. Logothetis, and Andreas S. Tolias. 2010. “Decorrelated Neuronal Firing in Cortical Microcircuits.” *Science* 327 (5965): 584–87.

Friston, K. J., L. Harrison, and W. Penny. 2003. “Dynamic Causal Modelling.” *NeuroImage* 19 (4): 1273–1302.

Glasser, Matthew F., Timothy S. Coalson, Emma C. Robinson, Carl D. Hacker, John Harwell, Essa Yacoub, Kamil Ugurbil, et al. 2016. “A Multi-Modal Parcellation of Human Cerebral Cortex.” *Nature* 536 (7615): 171–78.

Hennequin, Guillaume, Yashar Ahmadian, Daniel B. Rubin, Máté Lengyel, and Kenneth D. Miller. 2018. “The Dynamical Regime of Sensory Cortex: Stable Dynamics around a Single Stimulus-Tuned Attractor Account for Patterns of Noise Variability.” *Neuron* 98 (4): 846–60.e5.

Ito, Takuya, Kaustubh R. Kulkarni, Douglas H. Schultz, Ravi D. Mill, Richard H. Chen, Levi I. Solomyak, and Michael W. Cole. 2017. “Cognitive Task Information Is Transferred between Brain Regions via Resting-State Network Topology.” *Nature Communications* 8 (1): 1027.

Litwin-Kumar, Ashok, and Brent Doiron. 2012. “Slow Dynamics and High Variability in Balanced Cortical Networks with Clustered Connections.” *Nature Neuroscience* 15 (11): 1498–1505.

Logothetis, N. K., J. Pauls, M. Augath, T. Trinath, and A. Oeltermann. 2001. “Neurophysiological Investigation of the Basis of the fMRI Signal.” *Nature* 412 (6843): 150–57.

Ma, Ying, Mariel G. Kozberg, Sharon H. Kim, Mohammed A. Shaik, Dmitriy Timmerman, and Elizabeth M. C. Hillman. 2016. “Resting - State Hemodynamics Are Spatiotemporally Coupled to Synchronized and Symmetric Neural Activity in Excitatory Neurons.” *Proceedings of the National Academy of Sciences*.

https://doi.org/10.1073/pnas.1525369113.
Pinto, Lucas, Kanaka Rajan, Brian DePasquale, Stephan Y. Thiberge, David W. Tank, and Carlos D. Brody. 2019. “Task-Dependent Changes in the Large-Scale Dynamics and Necessity of Cortical Regions.” *Neuron*, August. https://doi.org/10.1016/j.neuron.2019.08.025.
Ponce-alvarez, Adrián, Biyu J. He, Patric Hagmann, and Gustavo Deco. 2015. “Task-Driven Activity Reduces the Cortical Activity Space of the Brain : Experiment and Whole-Brain Modeling,” 1–26.
Power, Jonathan D., Alexander L. Cohen, Steven M. Nelson, Gagan S. Wig, Kelly Anne Barnes, Jessica a. Church, Alecia C. Vogel, et al. 2011. “Functional Network Organization of the Human Brain.” *Neuron* 72 (4): 665–78.
Rosenbaum, Robert, Jonathan E. Rubin, and Brent Doiron. 2012. “Short-Term Synaptic Depression and Stochastic Vesicle Dynamics Reduce and Shape Neuronal Correlations.” *Journal of Neurophysiology* 109 (2): 475–84.
Ruff, Douglas A., and Marlene R. Cohen. 2014. “Attention Can Either Increase or Decrease Spike Count Correlations in Visual Cortex.” *Nature Neuroscience* 17 (11): 1591–97.
Schaefer, Alexander, Ru Kong, Evan M. Gordon, Timothy O. Laumann, Xi-Nian Zuo, Avram J. Holmes, Simon B. Eickhoff, and B. T. Thomas Yeo. 2018. “Local-Global Parcellation of the Human Cerebral Cortex from Intrinsic Functional Connectivity MRI.” *Cerebral Cortex* 28 (9): 3095–3114.
Silveira, Rava Azeredo da, and Michael J. Berry 2nd. 2014. “High-Fidelity Coding with Correlated Neurons.” *PLoS Computational Biology* 10 (11): e1003970.
